# Supplementary material for: Copper Foam as Active Catalysts for the Borylation of α, β-Unsaturated Compounds
Source: Int J Mol Sci. 2022 Jul 29;23(15):8403. doi: 10.3390/ijms23158403 (PMC9368805; doi:10.3390/ijms23158403)
Supplement: Supplementary file 1 [file ijms-23-08403-s001.zip › ijms-1848343-supplementary.pdf]

Supplementary Information

Copper Foam as Active Catalysts for the Borylation of α, β-Unsaturated Compounds

Kewang Zheng [1,2], Miao Liu [1], Zhifei Meng [1], Zufeng Xiao [1], Fei Zhong [1,*], Wei Wang [1,*] and Caiqin Qin [1]

[1] College of Chemistry and Materials Science, Hubei Engineering University, Xiaogan 432000, China; kewang1104@126.com (K.Z.); liumiao0727@163.com (M.L.); meng16301@163.com (Z.M.); chemhbeu@163.com (Z.X.); qincq@hbeu.edu.cn (C.Q.)

[2] Hubei Key Laboratory of Biological Resources and Environmental Biotechnology, Wuhan University, Wuhan 430079, China

* Correspondence: zhong4536188@hbeu.edu.cn (F.Z.); weiwang@hbeu.edu.cn (W.W.)

Table of Contents

1. Table **S1** The atomic percentage of copper foam 1

2. Characterization data for products (**2a, 3aa, 3a-3y**) 2

3. Figures **S1–S27** ^{1}H NMR and ^{13}C NMR spectra of products (**2a, 3aa, 3a-3y**) 10

Table S1. The atomic percentage of copper foam.

	copper foam	copper foam (after one reaction),	copper foam (after one reaction),
O (atomic percentage)	2.37	1.52	1.46
Cu (atomic percentage)	97.83	98.48	98.54

2. **Characterization data for products**

Most of adducts are literature-known [43-46] and obtained characterization data for these compounds is in full agreement with reported data.

2a 3-(4-methoxyphenyl)-1-phenyl-3-(4,4,5,5-tetramethyl-1,3,2-dioxaborolan-2-yl)propan-1-one

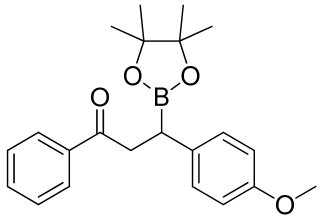

^{1}H NMR (400 MHz, $CDCl_3$, ppm) δ = 7.97-7.95 (m, 2H), 7.55-7.50 (m, 1H), 7.44-7.40 (m, 2H), 7.25-7.22 (m, 2H), 6.87-6.83 (m, 2H), 3.77 (s, 3H), 3.56-3.48 (dd, *J* = 18.2 Hz, 10.8 Hz, 1H), 3.42-3.37(dd, *J* = 18.2 Hz, 5.2 Hz, 1H), 3.77-3.73(dd, *J* = 10.8 Hz, 5.2 Hz, 1H), 1.26 (s, 6H), 1.18(s, 6H).

^{13}C NMR (100 MHz, $CDCl_3$, ppm) δ = 199.61, 157.45, 136.56, 133.62, 132.78, 129.13, 128.33, 127.86, 113.80, 83.15, 55.00, 43.43, 24.44, 24.40.

3aa 3-Hydroxy-3-(4-methoxyphenyl)-1-phenylpropan-1-one

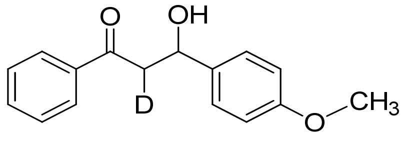

^{1}H NMR (400 MHz, $CDCl_3$, ppm) δ = 7.97-7.94 (m, 2H), 7.61-7.57 (m, 1H), 7.49-7.45 (m, 2H), 7.38-7.35 (m, 2H), 6.93-6.89 (m, 2H), 5.30-5.28 (d, *J* = 9.4 Hz 1H), 3.81 (s, 3H), 3.59 (s, 1H), 3.37-3.32 (s, 2H).

^{13}C NMR (100 MHz, $CDCl_3$, ppm) δ = 200.30, 200.24, 159.05, 136.52, 136.51, 135.04, 133.60, 128.66, 128.11, 126.99, 113.88, 77.32, 77.00, 76.68, 69.62, 55.27, 47.29, 47.13, 46.94, 46.75.

3a 3-Hydroxy-3-(4-methoxyphenyl)-1-phenylpropan-1-one

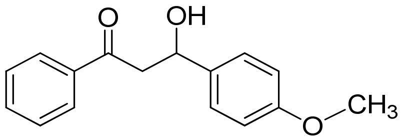

^1H NMR (400 MHz, $CDCl_3$, ppm) δ = 7.97-7.94 (m, 2H), 7.61-7.56 (tt, *J* = 7.4 Hz, 1.4Hz, 1H), 7.49-7.45 (m, 2H), 7.38-7.35 (m, 2H), 6.93-6.90 (m, 2H), 5.32-5.28 (m, 1H), 3.81 (s, 3H), 3.53-3.52 (d, *J* = 3.0 Hz, 1H), 3.37-3.35 (m, 2H).

^{13}C NMR (100 MHz, $CDCl_3$, ppm) δ = 200.01, 158.97, 136.53, 135.14, 133.44, 128.55, 128.04, 126.93, 113.80, 69.56, 55.17, 47.25.

3b. 3-Hydroxy-1-phenyl-3-(*p*-tolyl)propan-1-one

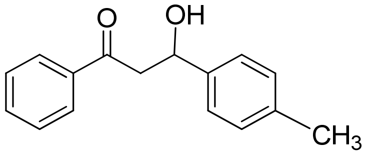

^1H NMR (400 MHz, $CDCl_3$, ppm) δ =7.97-7.94 (m, 2H), 7.61-7.57(tt, *J* = 7.4 Hz, 1.4Hz 1H), 7.49-7.44 (m, 2H), 7.35-7.33 (d, *J* =8.0 Hz, 2H), 7.20-7.18(d, *J* = 7.8 Hz, 2H), 5.34-5.30 (m, 1H), 3.54 (d, *J* = 2.7 Hz, 1H), 3.38-3.32 (m, 2H), 2.36 (s, 3H).

^{13}C NMR (100 MHz, $CDCl_3$, ppm) δ = 200.09, 140.00, 137.25, 136.56, 133.51, 129.15, 128.61, 128.09, 125.64, 69.83, 47.34, 21.06.

3c 3-Hydroxy-1-phenyl-3-(4-(trifluoromethyl)phenyl)propan-1-one

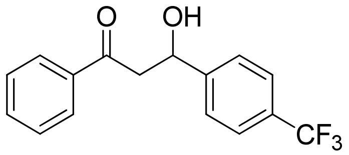

^{1}H NMR (400 MHz, $CDCl_3$, ppm) δ = 7.96-7.94(m, 2H), 7.65-7.56(m, 5H), 7.50-7.46(t, *J* =8.0 Hz, 2H), 5.43-5.40(q, *J* =4.0 Hz, 1H), 3.79(s,1H), 3.39-3.35 (m, 2H).

^{13}C NMR (100 MHz, $CDCl_3$, ppm) δ = 199.78, 146.87, 136.31, 133.87, 130.02-130.01(d, *J* =1.6 Hz), 129.72-129.681(d, *J* =3.6 Hz), 128.75, 128.11, 126.02, 125.56-125.45(dd, *J* =7.4 Hz, 3.4 Hz), 69.46, 47.17.

3d 3-(4-fluorophenyl)-3-hydroxy-1-phenylpropan-1-one

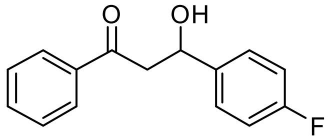

^{1}H NMR (400 MHz, $CDCl_3$, ppm) δ = 7.97-7.94 (m, 2H) 7.62-7.58 (m, 1H), 7.49-7.45(t, *J* = 7.8 Hz, 2H), 7.43-7.40(m, 2H), 7.09- 7.04(m, 2H), 5.35-5.31(m, 1H), 3.62-3.61 (d, *J* = 2.8 Hz, 1H), 3.36-3.34(m, 2H).

^{13}C NMR (100 MHz, $CDCl_3$, ppm) δ = 200.00, 163.42, 160.98, 138.72-138.69 (d, *J* = 20.8 Hz), 136.46, 133.70, 128.70, 128.12, 127.46- 127.38(d, *J* = 80.4Hz), 115.45-115.24(d, *J* = 212.0Hz), 69.43, 47.32.

3e 3-(4-chlorophenyl)-3-hydroxy-1-phenylpropan-1-one

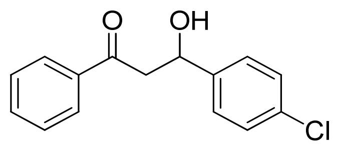

^{1}H NMR (400 MHz, $CDCl_3$, ppm) δ = 7.96-7.94 (m, 2H), 7.62-7.58(m, 1H), 7.49-7.46 (t, *J* = 8.0Hz, 2H), 7.39-7.34(m, 4H), 5.33 (dt, *J* = 11.2, 3.6Hz, 1H), 3.64 (dd, *J* = 5.4 Hz, 3.0 Hz, 1H), 3.40-3.25 (m, 2H).

^{13}C NMR (100 MHz, $CDCl_3$, ppm) δ = 199.84, 141.44, 136.36, 133.72, 133.25, 128.69, 128.62, 128.09, 127.12, 69.34, 47.19.

3f 3-(3-bromophenyl)-3-hydroxy-1-phenylpropan-1-one

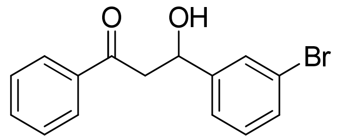

^{1}H NMR (400 MHz, $CDCl_3$, ppm) δ = 7.96-7.93(m, 2H), 7.62-7.58 (m, 2H), 7.49-7.45 (t, *J* = 7.8Hz, 2H), 7.43-7.41(dd, *J* = 8.0 Hz, 1.2 Hz, 1H), 7.36-7.34(d, *J* = 8.0 Hz, 1H), 7.26-7.22 (t, *J* = 7.8Hz, 1H), 5.33-5.29(m, 1H), 3.75(s, 1H), 3.35-3.33(m, 2H).

^{13}C NMR (100 MHz, $CDCl_3$, ppm) δ = 99.81, 145.21, 136.28, 133.77, 130.65, 130.09, 128.86, 128.71, 128.11, 124.34, 122.65, 69.29, 47.19.

3g 3-(4-(benzyloxy)phenyl)-3-hydroxy-1-phenylpropan-1-one

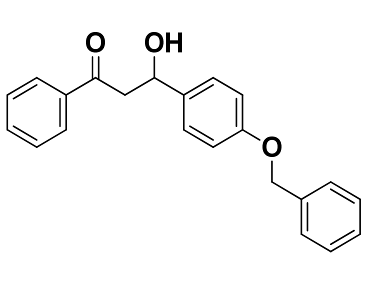

^1H NMR (400 MHz, $CDCl_3$, ppm) δ = 7.97-7.95(m, 2H), 7.61-7.57(m, 1H), 7.49-7.31(m, 9H), 7.01-6.97 (m, 2H), 5.32- 5.29(dd, *J* = 7.4 Hz, 4.8 Hz, 1H), 5.08(s, 2H), 3.54(s, 1H), 3.38-3.36(m, 2H),.

^{13}C NMR (100 MHz, $CDCl_3$, ppm) δ = 200.24, 158.31, 136.95, 136.57, 135.37, 133.60, 128.68, 128.56, 128.13, 127.93, 127.42, 127.03, 114.90, 70.03, 69.67, 47.30.

3h 3-Hydroxy-1,3-diphenylpropan-1-one

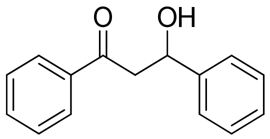

^1H NMR (400 MHz, $CDCl_3$, ppm) δ = 7.97-7.95 (m, 2H), 7.61-7.57 (tt, *J* = 7.2Hz, 1.2 Hz, 1H), 7.49-7.44 (m, 4H), 7.40-7.37 (t, *J* = 7.4 Hz, 2H), 7.32-7.29 (m, 1H), 5.37-5.34 (t, *J* = 2.2 Hz, 1H), 3.61 (s, 1H), 3.39-3.31 (m, 2H).

^{13}C NMR (100 MHz, $CDCl_3$, ppm) δ = 199.88, 142.96, 136.47, 133.44, 128.53, 128.39, 128.03, 127.49, 125.64, 69.87, 47.29.nn

3i 3-hydroxy-1-(4-methoxyphenyl)-3-phenylpropan-1-one

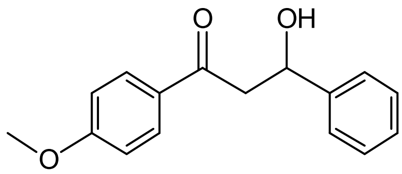

^1H NMR (400 MHz, $CDCl_3$, ppm) δ = 7.95-7.92 (m, 2H), 7.46-7.43 (m, 2H), 7.40-7.36 (m, 2H), 7.32-7.28(tt, *J* = 6.4 Hz, 1.4 Hz, 1H), 6.95- 6.92 (m, 2H), 5.34- 5.31 (m, 1H), 3.87(s, 3H), 3.77(s, 1H), 3.33- 3.30(m, 2H).

^{13}C NMR (100 MHz, $CDCl_3$, ppm) δ = 198.73, 163.92, 143.04, 130.47, 129.63, 128.51, 127.57, 125.73, 113.83, 70.14, 55.49, 46.90.

3j 3-Hydroxy-3-phenyl-1-(*p*-tolyl)propan-1-one

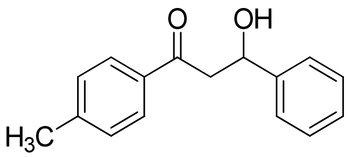

^1H NMR (400 MHz, $CDCl_3$, ppm) δ = 7.87-7.85 (m, 2H), 7.46 – 7.43(m, 2H), 7.40-7.36 (m, 2H), 7.32-7.30 (m, 1H), 7.27-7.25 (d, *J* = 7.6 Hz,4H), 5.34 (m, 1H), 3.66-3.65 (d, *J* = 2.8 Hz, 1H), 3.35-3.34 (m, 2H), 2.42 (s, 3H).

^{13}C NMR (100 MHz, $CDCl_3$, ppm) δ = 199.82, 144.55, 142.98, 134.08, 129.34, 128.50, 128.24, 127.58, 125.72, 70.05, 47.18, 21.66.

3k 1-(4-fluorophenyl)-3-hydroxy-3-phenylpropan-1-one

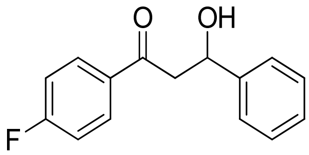

^1H NMR (400 MHz, $CDCl_3$, ppm) δ = 8.01-7.96 (m, 2H), 7.45-7.43 (m, 2H), 7.41-7.35 (m, 2H), 7.33-7.29 (m, 1H), 7.17-7.11 (m, 2H), 5.37-5.33 (qui, *J* = 3.6 Hz, 1H), 3.48-3.48 (d, *J* = 3.0 Hz, 1H), 3.36-3.34(m, 2H).

^{13}C NMR (100 MHz, $CDCl_3$, ppm) δ = 198.40, 167.29-164.75(d, *J* = 254.2Hz), 142.83, 133.04-133.01(d, *J* = 2.0 Hz), 130.88-130.78(d, *J* = 9.4 Hz), 128.57, 127.71, 125.69, 115.93-115.71(d, *J* = 21.8 Hz), 69.99, 47.33.

3l 3-Hydroxy-1,3-bis(4-methoxyphenyl)propan-1-one

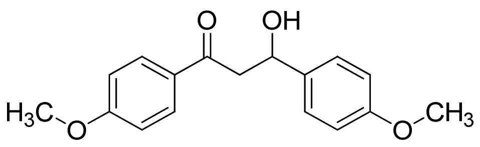

^{1}H NMR (400 MHz, $CDCl_3$, ppm) δ = 7.94-7.90 (m, 2H), 7.37-7.34 (m, 2H), 6.94-6.88 (m, 4H), 5.28-5.25 (td, *J* = 6.2 Hz, 2.4 Hz, 1H), 3.86 (s, 3H), 3.80 (s, 3H), 3.72 (d, *J* = 2.4 Hz, 1H), 3.30-3.29 (d, *J* = 6.0 Hz, 2H).

^{13}C NMR (100 MHz, $CDCl_3$, ppm) δ =198.73, 163.84, 159.00, 135.25, 130.43, 129.66, 126.97, 113.84, 113.77, 69.75, 55.44, 55.23, 46.82.

3m 3-(4-bromophenyl)-3-hydroxy-1-(4-methoxyphenyl)propan-1-one

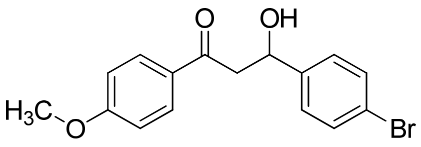

^{1}H NMR (400 MHz, $CDCl_3$, ppm) δ = 7.94-7.91 (m, 2H), 7.52-7.48 (m, 2H), 7.33-7.31 (m, 2H), 6.95-6.92 (m, 2H), 5.30 – 5.26 (m, 1H), 3.88 (s, 3H), 3.81-3.80 (d, *J* = 2.6 Hz, 1H), 3.30-3.25(m, 2H).

^{13}C NMR (100 MHz, $CDCl_3$, ppm) δ = 198.36, 163.97, 142.10, 131.51, 130.44, 129.42, 127.46, 121.25, 113.83, 69.50, 55.47, 46.64.

3n 3-(2-chlorophenyl)-3-hydroxy-1-(4-methoxyphenyl)propan-1-one

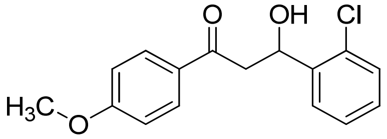

^{1}H NMR (400 MHz, $CDCl_3$, ppm) δ = 7.95-7.92(m, 2H), 7.73-7.71 (dd, *J* = 7.8 Hz, 1.8 Hz, 1H), 7.35-7.31 (t, *J* = 8.8 Hz, 2H), 7.25-7.20 (td, *J* = 7.6 Hz, 1.8 Hz, 1H),

6.94-6.91 (m, 2H), 5.67-5.64 (dt, *J* =9.6 Hz, 2.4Hz, 1H), 4.06 (s, 1H), 3.86 (s, 3H), 3.54-3.49 (m, 1H), 3.07 (dd, *J* = 17.4 Hz, 9.6 Hz, 1H).

^{13}C NMR (100 MHz, $CDCl_3$, ppm) δ =198.80, 163.94, 140.42, 131.09, 130.50, 129.43, 129.23, 128.45, 127.24, 127.19, 113.80, 66.91, 55.47, 44.77.

3o 3-(4-fluorophenyl)-3-hydroxy-1-(p-tolyl)propan-1-one

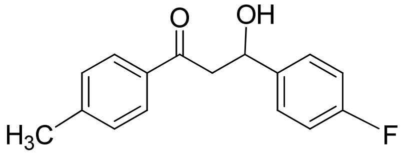

^{1}H NMR (400 MHz, $CDCl_3$, ppm) δ = 7.86-7.84 (m, 2H), 7.43-7.39 (m, 2H), 7.27-7.25 (d, *J* = 8.0 Hz, 3H), 7.09-7.03 (m, 2H), 5.33-5.29 (m, 1H), 3.71-3.71 (d, *J* = 2.8 Hz, 1H), 3.33-3.30 (m, 2H), 2.42 (s, 3H).

^{13}C NMR (100 MHz, $CDCl_3$, ppm) δ = 199.59, 163.32-160.88 (d, *J* = 244.0 Hz), 144.61, 138.80-138.77 (d, *J* = 244.0 Hz), 133.96, 129.32, 128.20, 127.42-127.34(d, *J* = 8.0 Hz), 115.34- 115.13(d, *J* = 21.2 Hz), 69.42, 47.09, 21.60.

3p 3-(4-chlorophenyl)-1-(4-fluorophenyl)-3-hydroxypropan-1-one

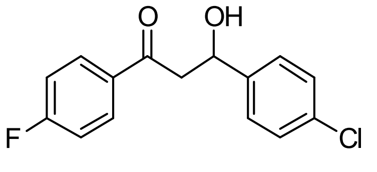

^{1}H NMR (400 MHz, $CDCl_3$, ppm) δ = 7.99-7.96 (dd, *J* = 8.4Hz, 5.4 Hz, 2H), 7.38-7.33 (m, 4H), 7.16-7.12 (t, *J* = 8.4 Hz, 2H), 5.33-5.30 (m, 1H), 3.60 (s, 1H), 3.31-3.30 (m, 2H).

^{13}C NMR (100 MHz, $CDCl_3$, ppm) δ = 198.21, 167.37-164.82(d, *J* = 254.6 Hz), 141.29, 133.37, 132.85-132.82 (d, *J* = 3.0 Hz), 130.88-130.79(d, *J* = 9.4 Hz), 128.70, 127.11, 116.01-115.80 (d, *J* = 21.8 Hz), 69.34, 47.17.

3q 3-Hydroxy-3-(naphthalen-1-yl)-1-phenylpropan-1-one

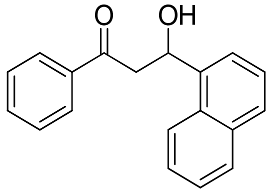

^{1}H NMR (400 MHz, $CDCl_3$, ppm) δ = 8.07-8.04 (m, 1H), 7.98-7.95 (m, 2H), 7.91-7.89 (m, 1H), 7.83-7.80 (dd, 8.6 Hz, 5.0 Hz, 2H), 7.61-7.57 (m, 1H), 7.54-7.49(m, 3H), 7.48-7.44(m, 2H), 6.18-6.15(dt, *J* = 9.2 Hz,, 2.6Hz, 1H), 3.75 (s, 1H), 3.60-3.54(dd, *J* = 18.0 Hz, 2.2 Hz, 1H), 3.52-3.45(q, *J* = 9.0 Hz, 1H)

^{13}C NMR (100 MHz, $CDCl_3$, ppm) δ = 200.31, 138.41, 136.47, 133.75, 133.68, 129.87, 129.03, 128.69, 128.17, 128.04, 126.22, 125.62, 125.54, 123.13, 122.76, 66.78, 46.77.

3r 4-hydroxy-4-(4-methoxyphenyl)butan-2-one

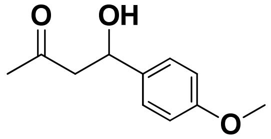

^{1}H NMR (400 MHz, $CDCl_3$, ppm) δ = 7.21- 7.19 (d, 8.4 Hz, 2H), 6.81- 6.79(d, 8.4 Hz, 2H), 5.03- 5.00 (dd, 9.2Hz, 2.8 Hz, 1H), 3.72(s, 3H), 3.20(s, 1H), 2.84-2.77(dd, *J* = 17.4Hz, 9.2 Hz, 1H), 2.72- 2.67 (m,1H), 2.11(s, 3H),.

^{13}C NMR (100 MHz, $CDCl_3$, ppm) δ = 209.12, 159.04, 134.87, 126.87, 113.84, 69.44, 55.23, 51.90, 30.73.

3s 4-(4-chlorophenyl)-4-hydroxybutan-2-one

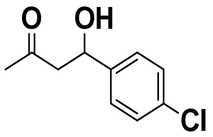

^1H NMR (400 MHz, $CDCl_3$, ppm) δ = 7.25-7.20(m,4H), 5.06-5.04(d, 8.6 Hz, 2H), 3.40-3.39(d, 3.2 Hz, 2H), 2.76-2.72(m,2H), 2.12(s,3H).

^{13}C NMR (100 MHz, $CDCl_3$, ppm) δ =208.91, 141.15, 133.31, 128.64, 127.00, 69.14, 51.76, 30.74.

3t 4-Hydroxy-4-phenylbutan-2-one

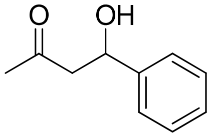

^1H NMR (400 MHz, $CDCl_3$, ppm) δ = 7.28-7.26 (d, *J* = 4.8 Hz,4H), 7.21-7.18 (m, 1H), 5.08-5.05 (dd, *J* = 9.0 Hz, 3.4 Hz, 1H), 3.26 (s, 1H), 2.84-2.77 (dd, *J* = 17.4Hz, 9.0 Hz, 1H), 2.75-2.69(dd, *J* = 17.4Hz, 3.4 Hz, 1H), 2.10 (s, 3H).

^{13}C NMR (100 MHz, $CDCl_3$, ppm) δ = 208.94, 142.74, 128.47, 127.60, 125.56, 69.80, 51.93, 30.69.

3u 1-Hydroxy-4,4-dimethyl-1-phenylpentan-3-one

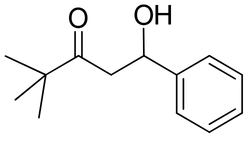

^{1}H NMR (400 MHz, $CDCl_3$, ppm) δ = 7.34-7.26(m, 4H), 7.25-7.22(m, 1H), 5.11-5.07(sext, *J* = 3.0 Hz, 1H), 3.55-3.54 (d, *J* = 3.0 Hz, 1H), 2.85- 2.84(d, *J* = 6.2Hz, 1H), 1.09(s, 9H).

^{13}C NMR (100 MHz, $CDCl_3$, ppm) δ = 216.71, 143.03, 128.41, 127.48, 125.60, 70.02, 45.43, 44.32, 26.08.

3v 3-hydroxy-3-phenylpropanenitrile

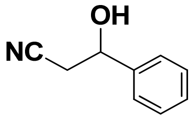

^{1}H NMR (400 MHz, $CDCl_3$, ppm) δ = 7.41-7.34(m, 5H), 5.06-5.03(sext, *J* = 3.2 Hz, 1H), 2.78-2.78(d, *J* = 1.6 Hz, 1H), .78-2.78(d, *J* = 0.8 Hz, 1H), 2.56-2.55(d, *J* = 3.6 Hz, 1H).

^{13}C NMR (100 MHz, $CDCl_3$, ppm) δ =140.96, 128.92, 128.84, 125.48, 117.25, 70.12, 27.92.

3w 3-Hydroxy-1,5-diphenylpent-4-en-1-one

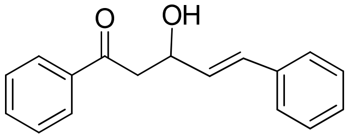

^{1}H NMR (400 MHz, $CDCl_3$, ppm) δ = 7.99-7.97 (m, 2H), 7.63-7.58 (m, 1H), 7.51-7.47 (m, 2H), 7.41-7.39 (m, 2H), 7.36 – 7.29 (m, 2H), 7.27-7.22 (m, 1H), 6.74-6.70 (dd, *J* = 16.0 Hz, 1.4 Hz, 1H), 6.35-6.30(dd, *J* = 16.0 Hz, 6.0 Hz, 1H), 4.98-4.95(m, 1H), 3.40 (s, 1H), 3.31-3.28 (m, 2H).

^{13}C NMR (100 MHz, $CDCl_3$, ppm) δ = 200.08, 136.59, 136.54, 133.66, 130.45, 130.21, 128.72, 128.56, 128.13, 127.71, 126.49, 77.32, 45.17, 29.69.

3x 3-hydroxy-1-phenyl-3-(thiophen-2-yl)propan-1-one

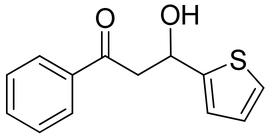

^{1}H NMR (400 MHz, $CDCl_3$, ppm) δ = 7.98-7.96 (m, 2H), 7.62-7.58 (m, 1H),7.50-7.46 (t, *J* = 7.6 Hz, 2H), 7.28-7.27 (m, 1H), 7.05-7.03(dd, *J* = 3.6 Hz,1.2 Hz, 1H), 7.00-6.98 (m, 1H), 5.63-5.59 (quint, *J* = 3.8 Hz 1H), 3.77-3.75 (m, 1H), 3.52-3.50 (m, 2H).

^{13}C NMR (100 MHz, $CDCl_3$, ppm) δ = 199.46, 146.66, 136.36, 133.65, 128.65, 128.09, 126.62, 124.59, 123.47, 66.35, 47.10.

3y 3-Hydroxy-3-(thiophen-2-yl)-1-(m-tolyl)propan-1-one

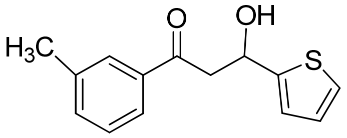

^{1}H NMR (400 MHz, Chloroform-*d*) δ 7.78-7.75 (m, 2H), 7.44-7.34 (m, 2H), 7.28-7.27 (dd, *J* = 5.0 Hz, 1.2 Hz, 1H) , 7.04-7.03 (m, 1H), 7.00-6.98 (dd, *J* = 5.0 Hz,, 3.4 Hz, 1H), 5.61-5.58 (t, *J* = 7.8 Hz, 1H), 3.81 (s, 1H), 3.50-3.49 (m, 2H), 2.42 (s, 3H).

^{13}C NMR (100 MHz, $CDCl_3$, ppm) δ =199.88, 146.63, 138.57, 136.40, 134.54, 128.63, 128.60, 126.67, 125.38, 124.66, 123.50, 66.46, 47.16, 21.33.

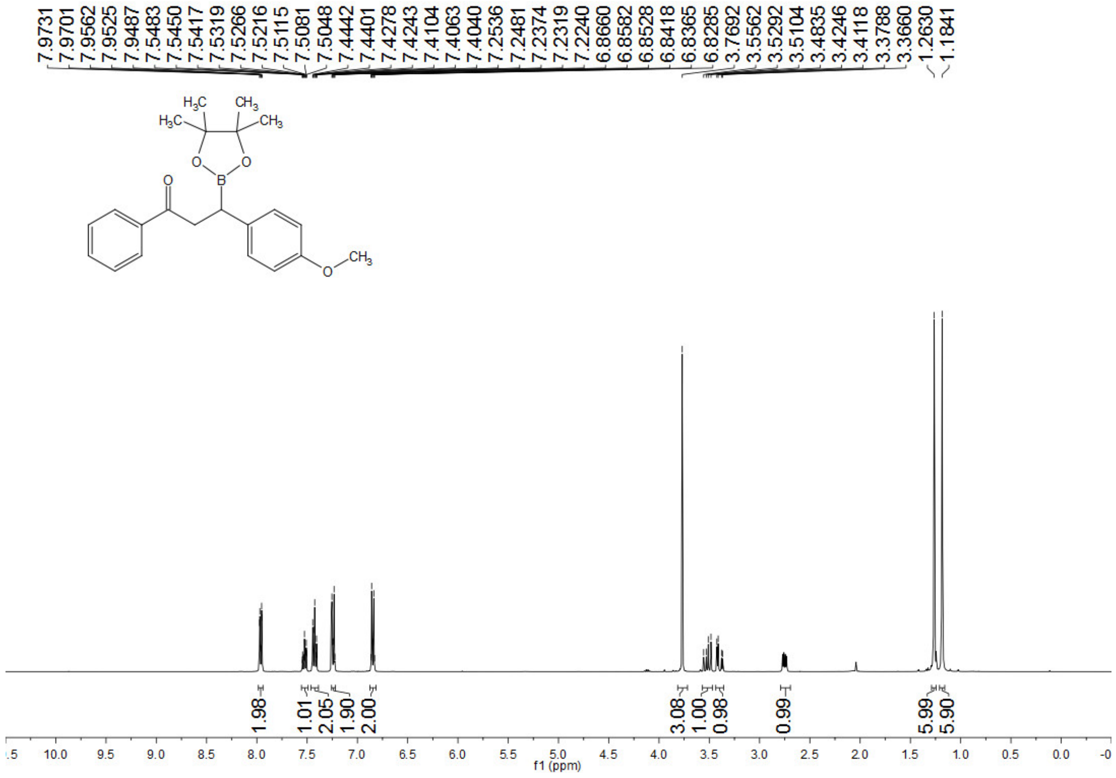

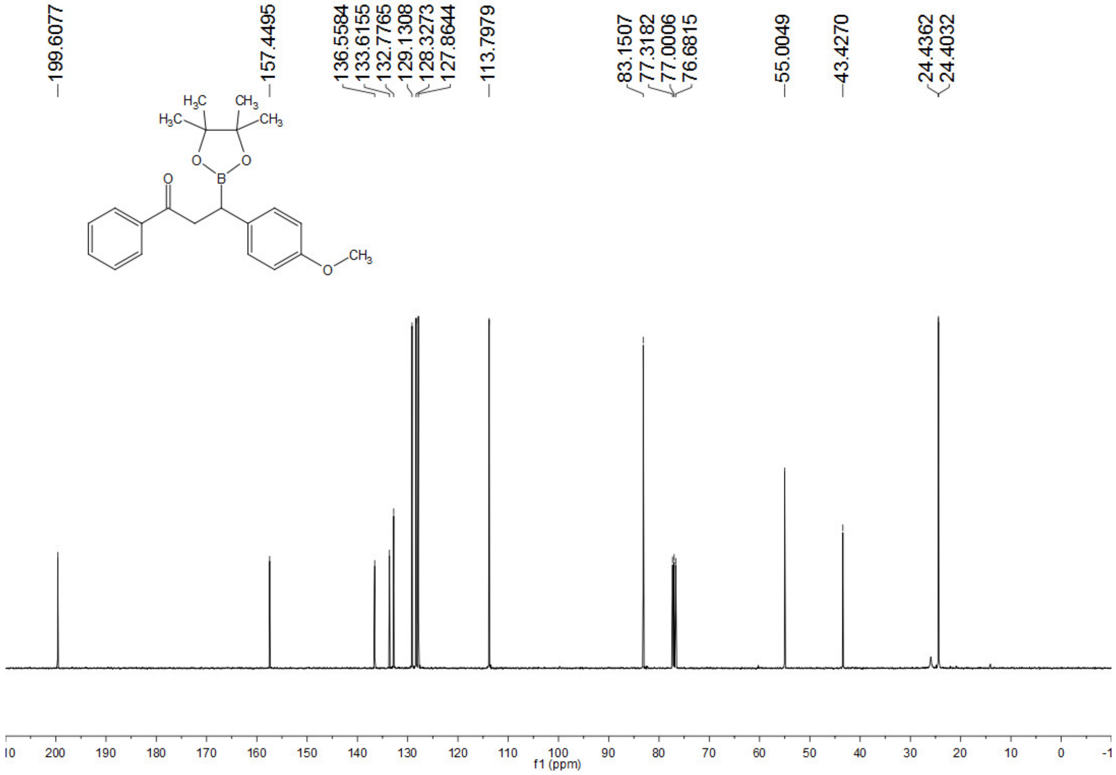

Figure S1. ^{1}H NMR and ^{13}C NMR spectra of **2a.**

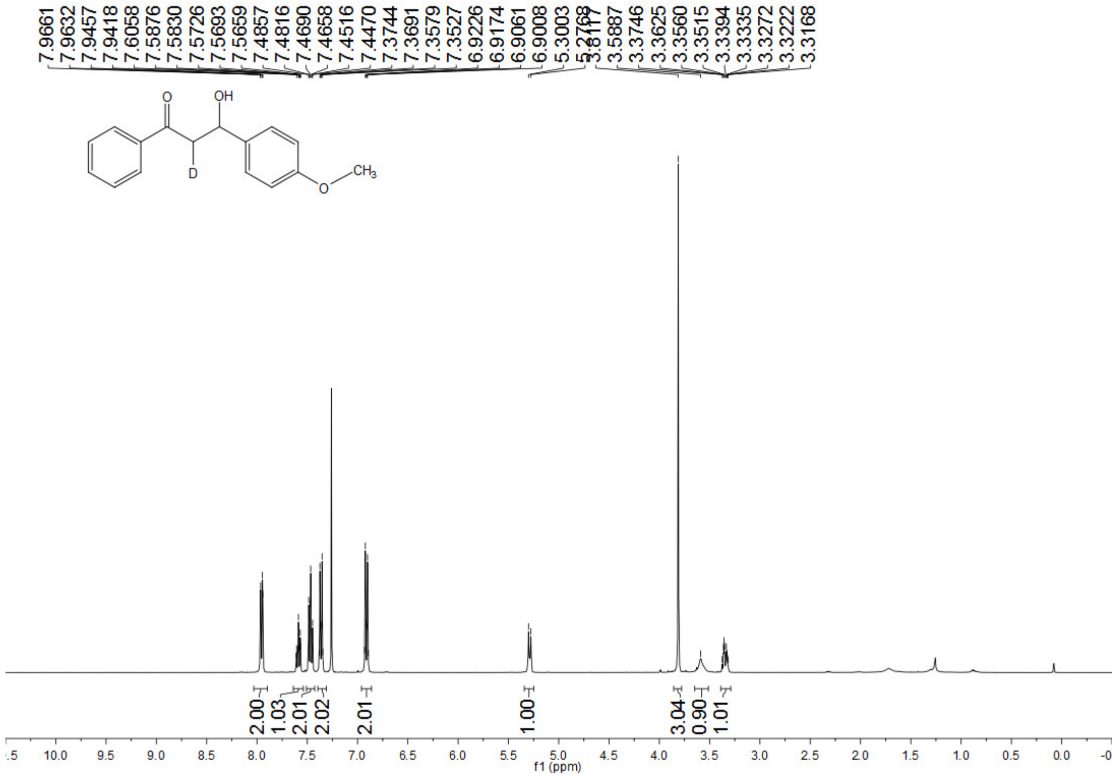

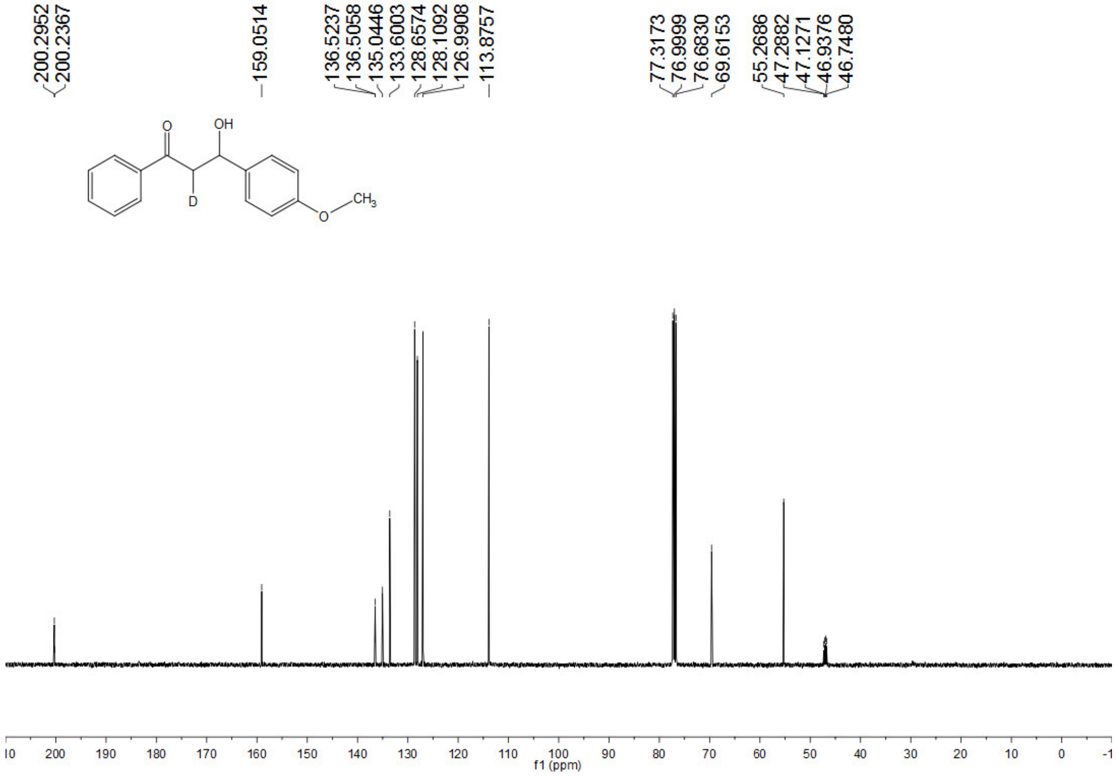

Figure S2. ^{1}H NMR and ^{13}C NMR spectra of **3aa.**

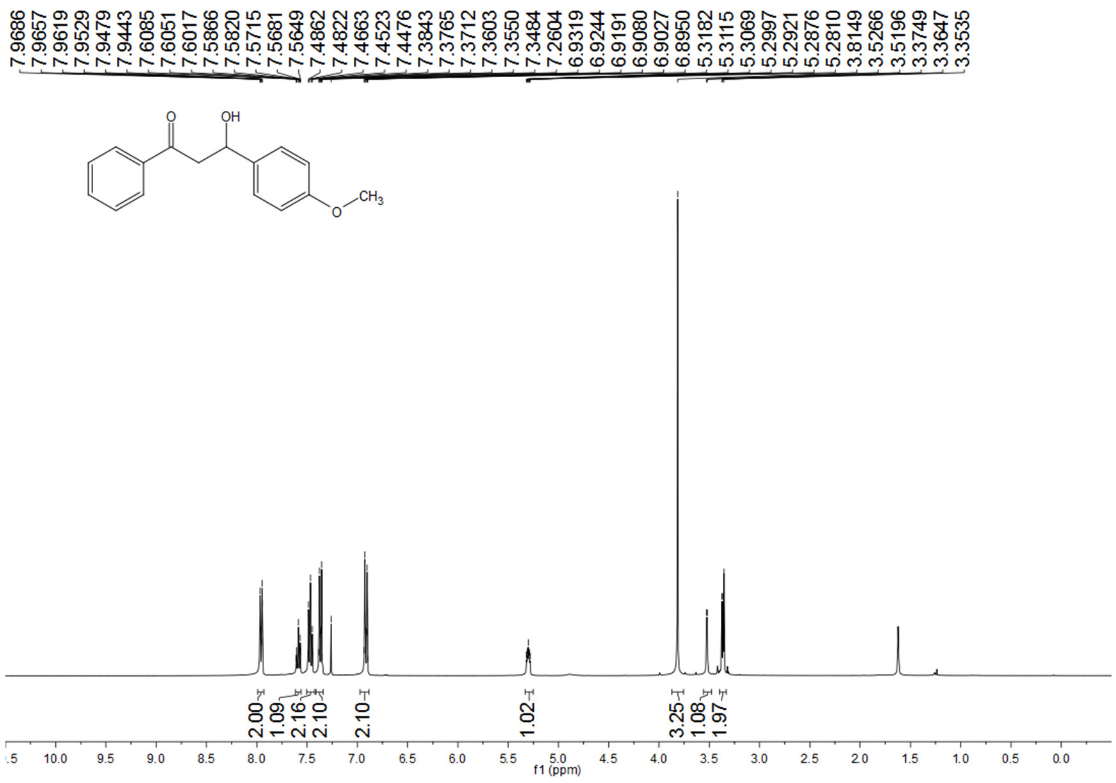

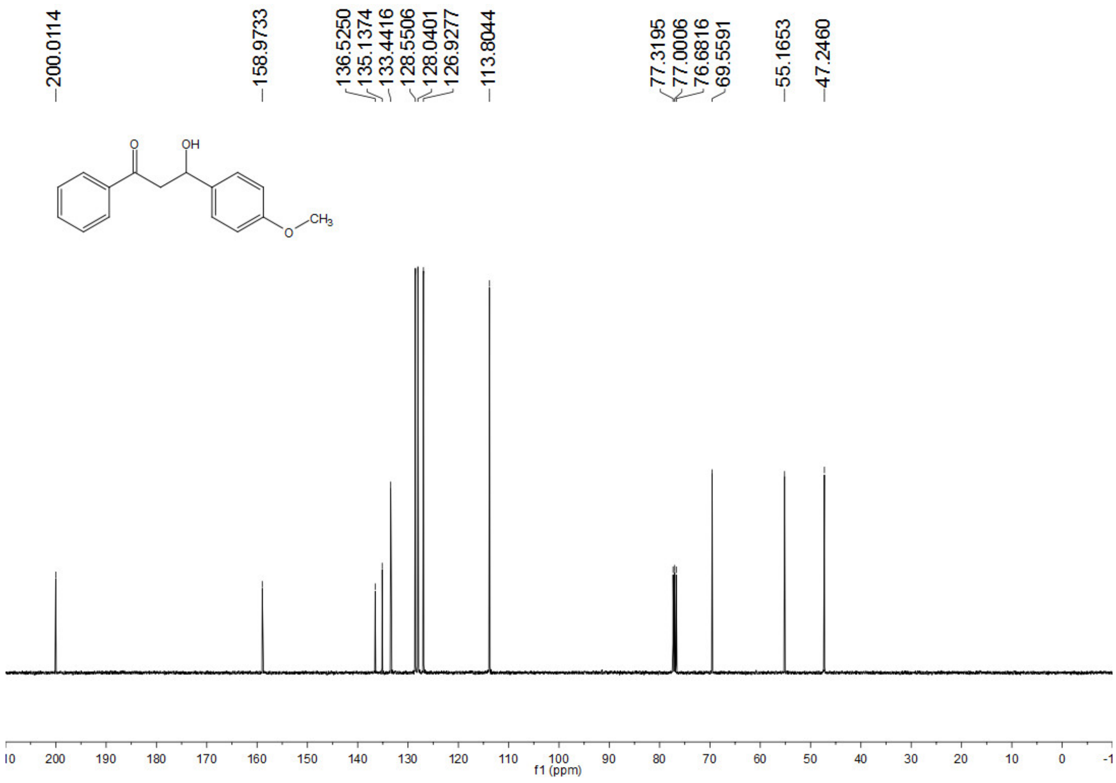

Figure S3. ^{1}H NMR and ^{13}C NMR spectra of **3a.**

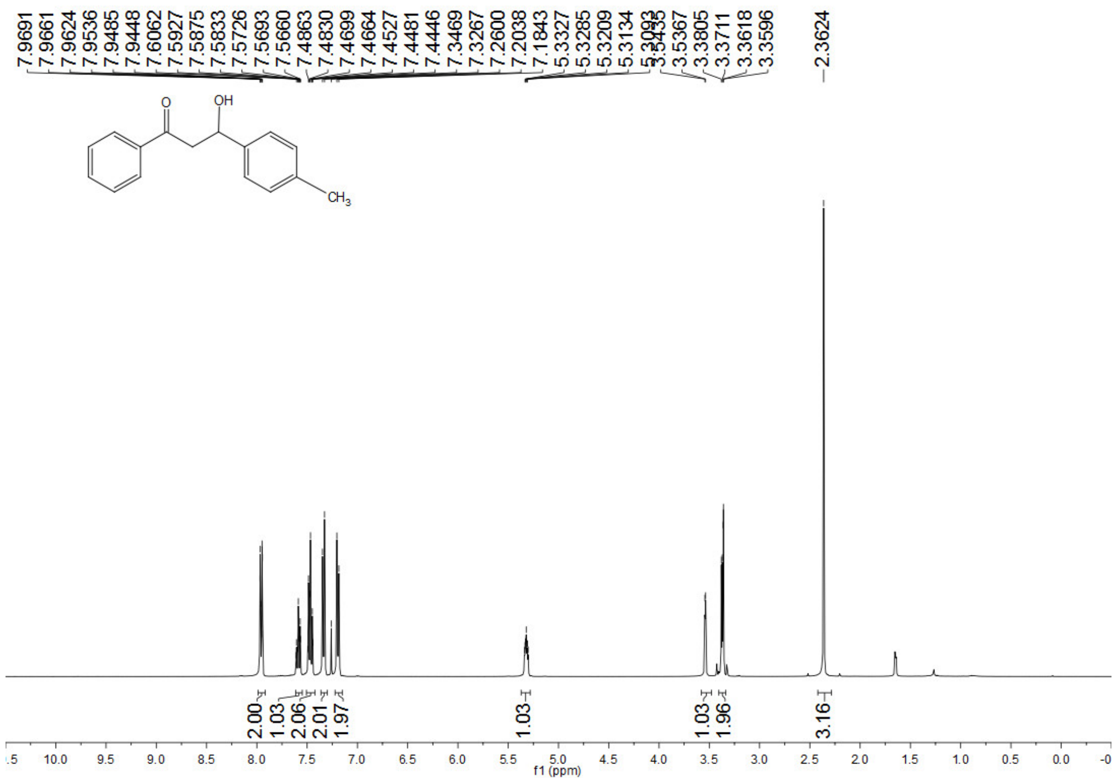

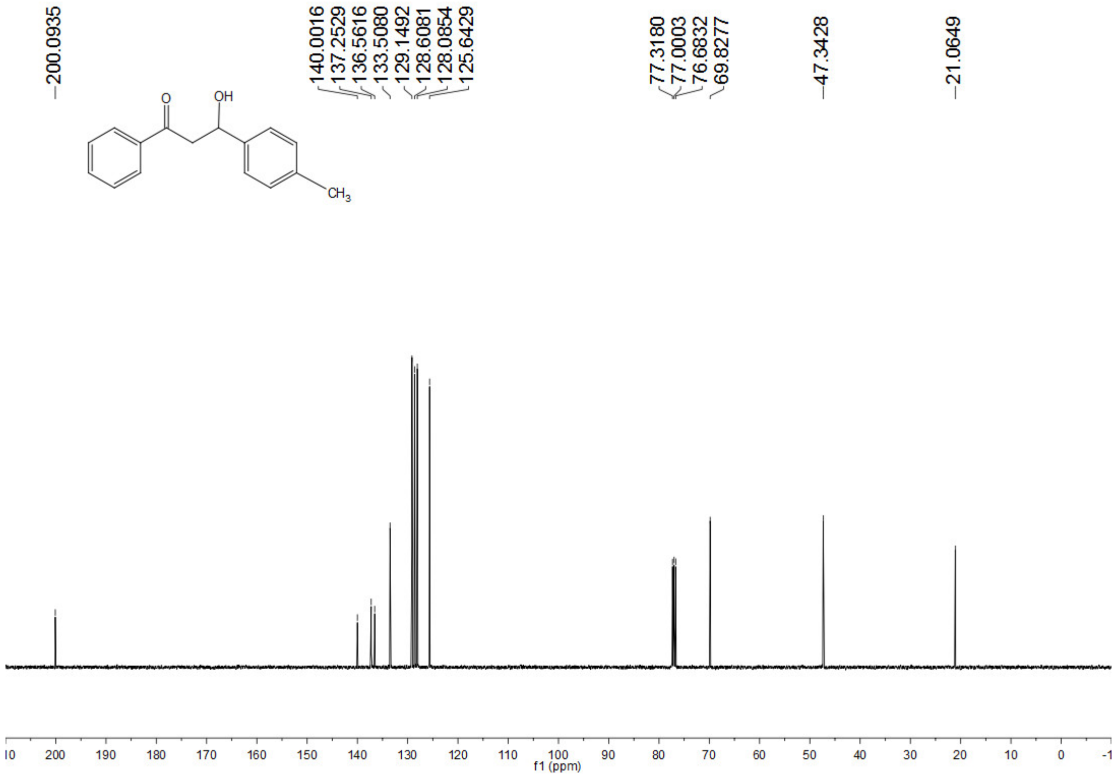

Figure S4. ^{1}H NMR and ^{13}C NMR spectra of **3b.**

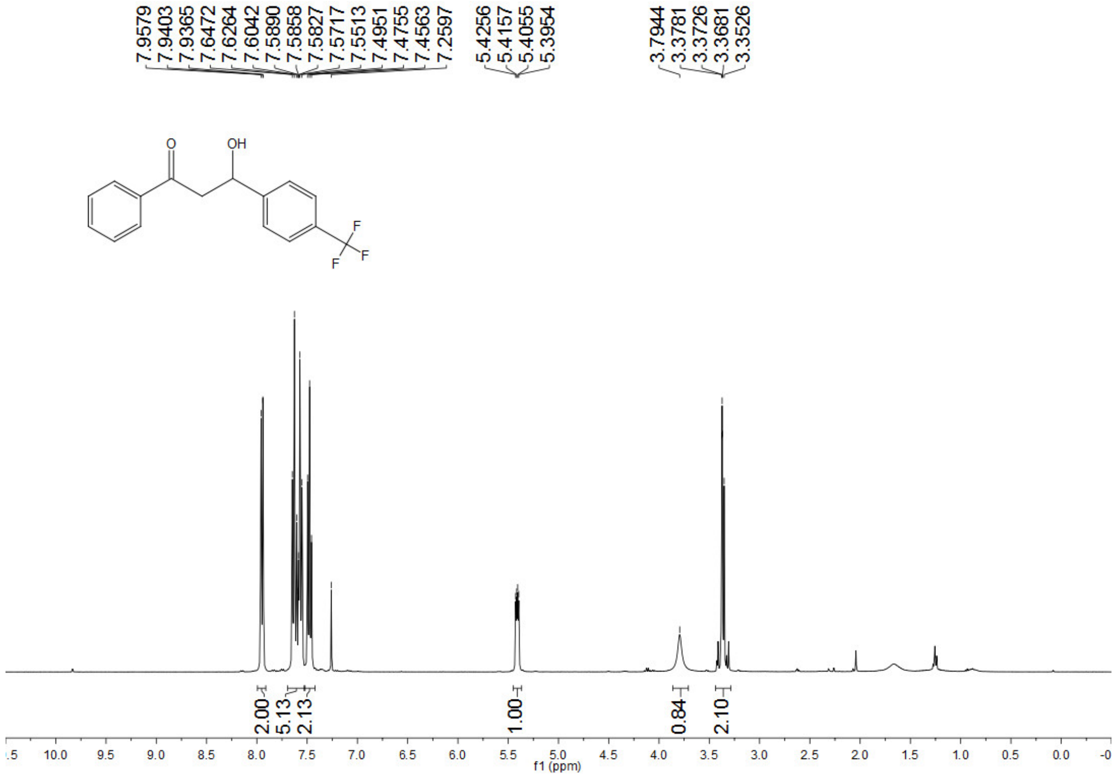

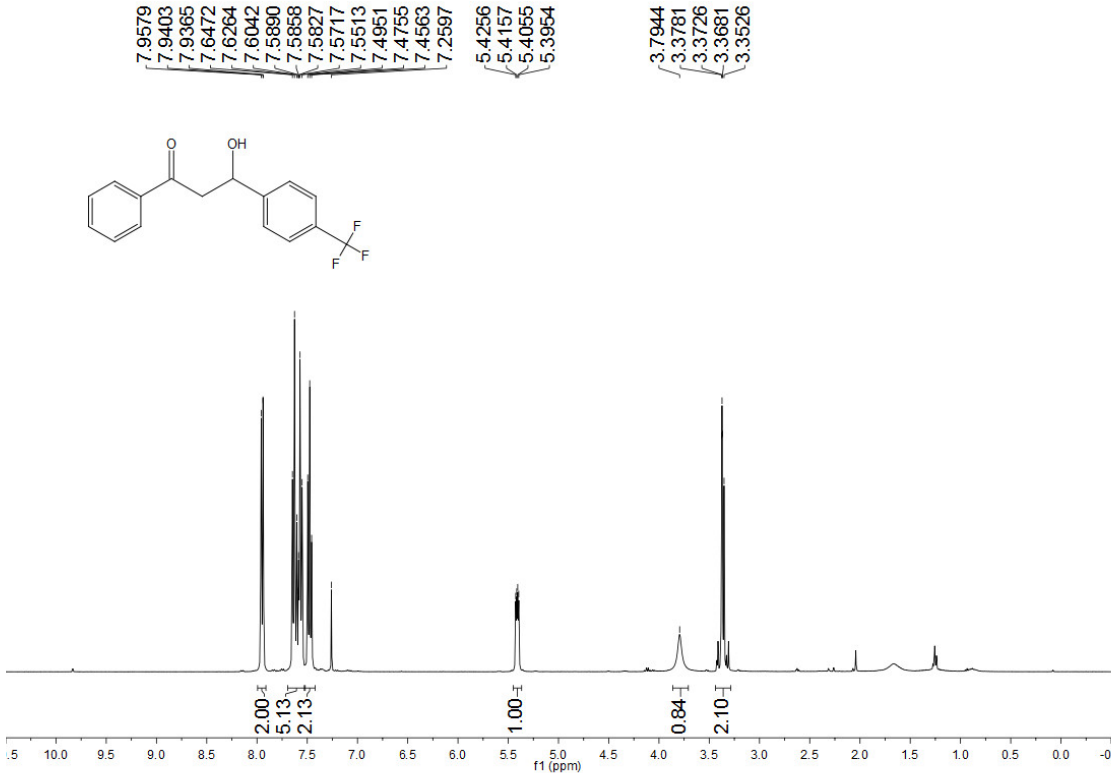

Figure S5. ^{1}H NMR and ^{13}C NMR spectra of **3c.**

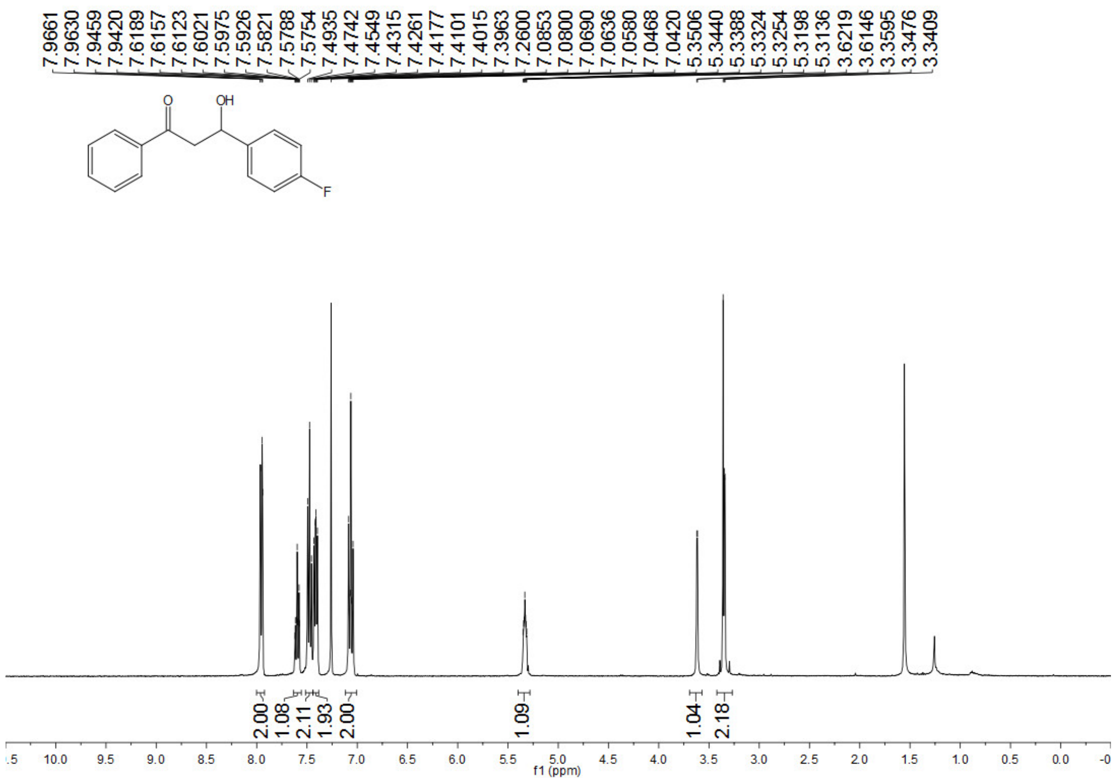

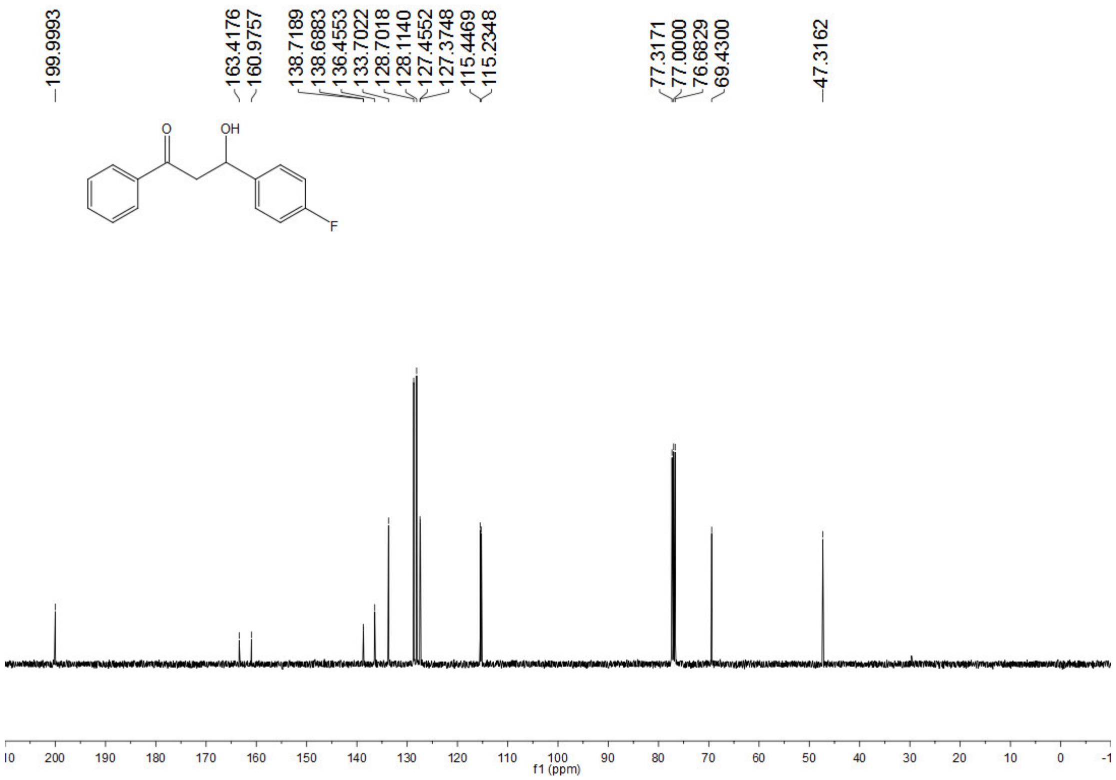

Figure S6. ^{1}H NMR and ^{13}C NMR spectra of **3d.**

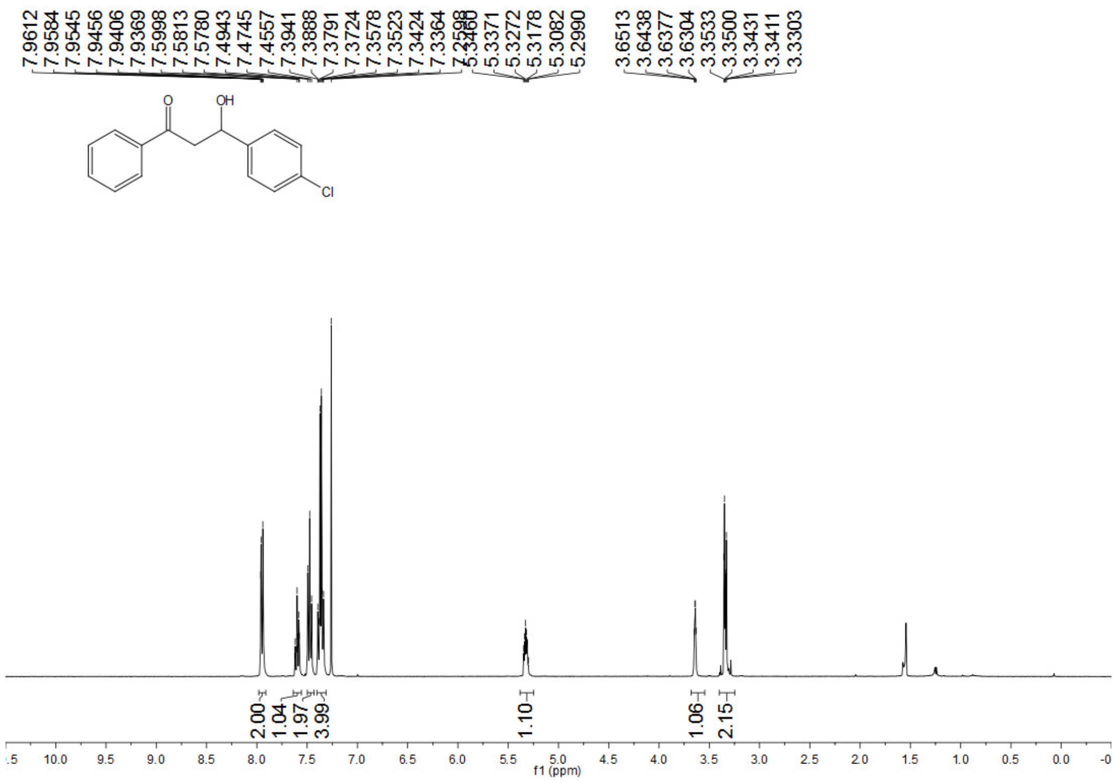

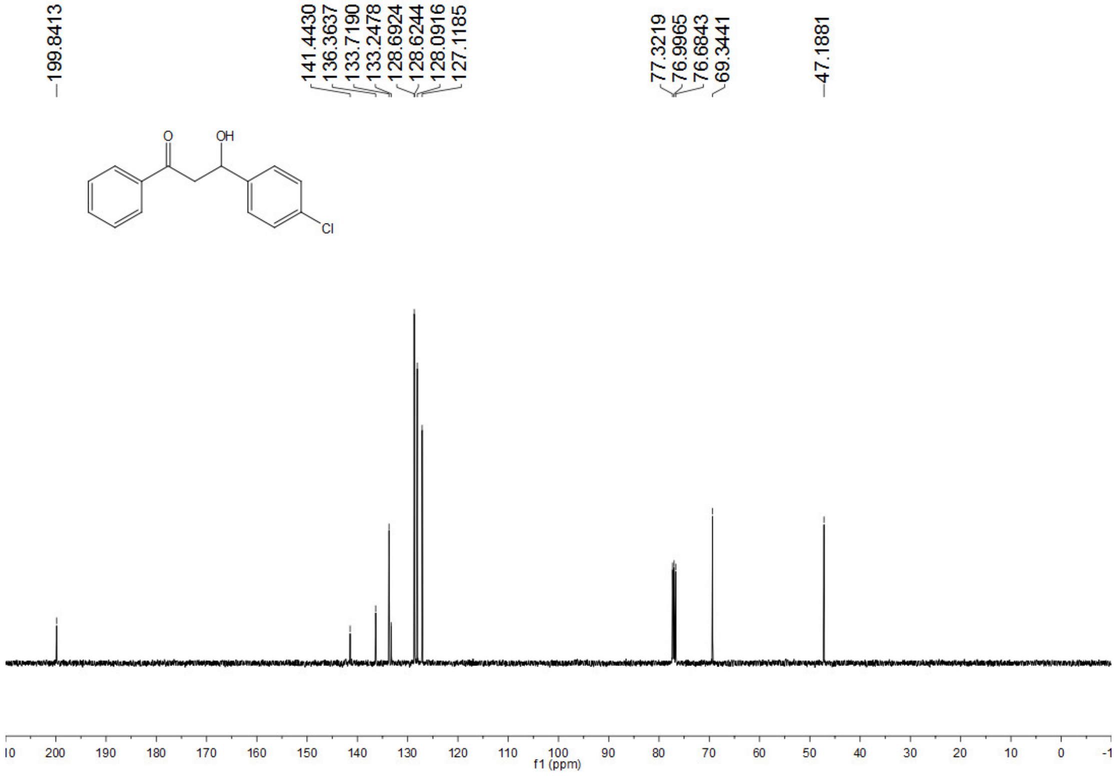

Figure S7. ^{1}H NMR and ^{13}C NMR spectra of **3e.**

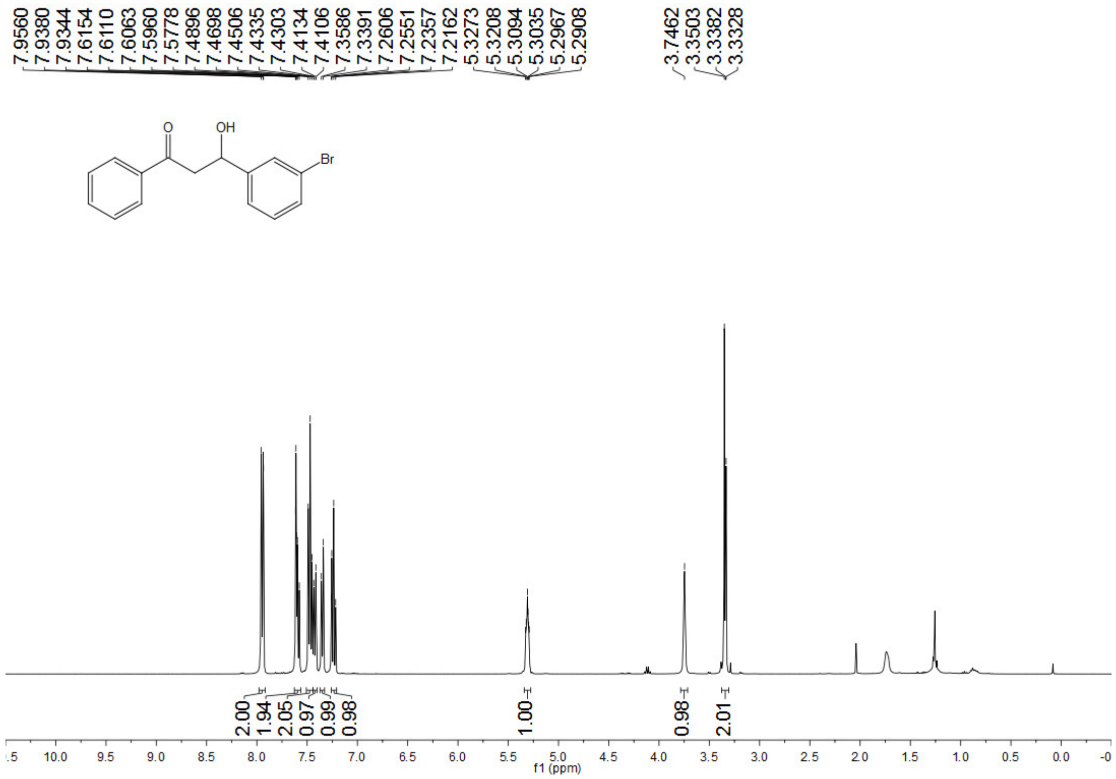

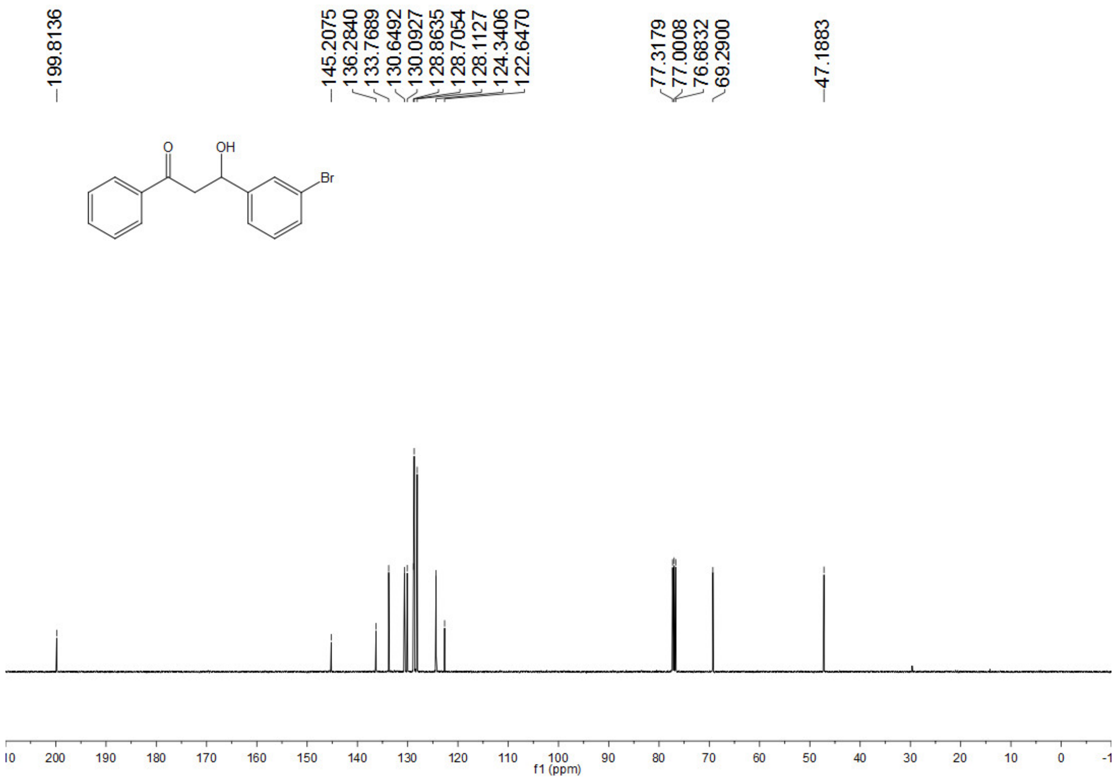

Figure S8. ^{1}H NMR and ^{13}C NMR spectra of **3f.**

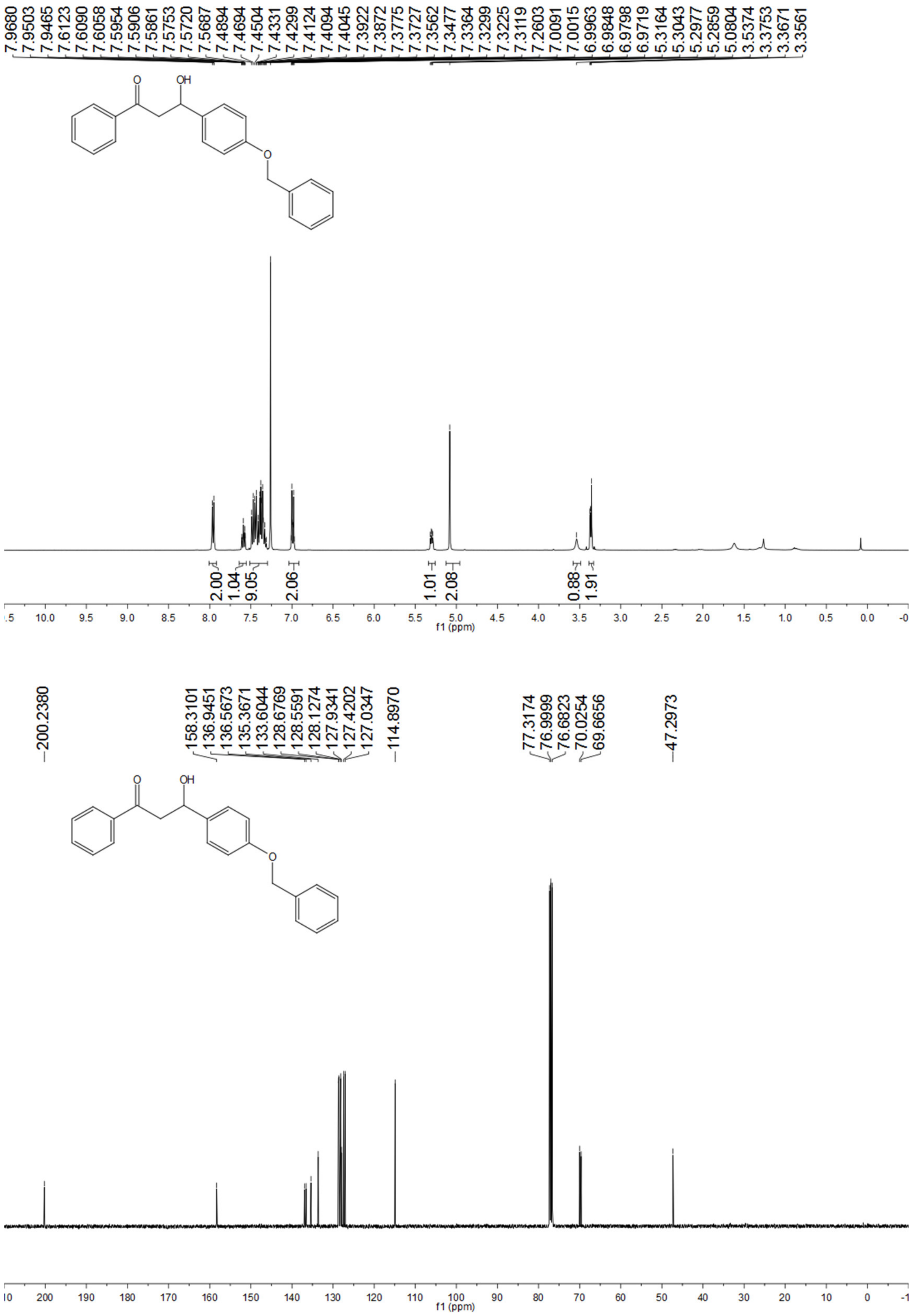

Figure S9. ^{1}H NMR and ^{13}C NMR spectra of **3g.**

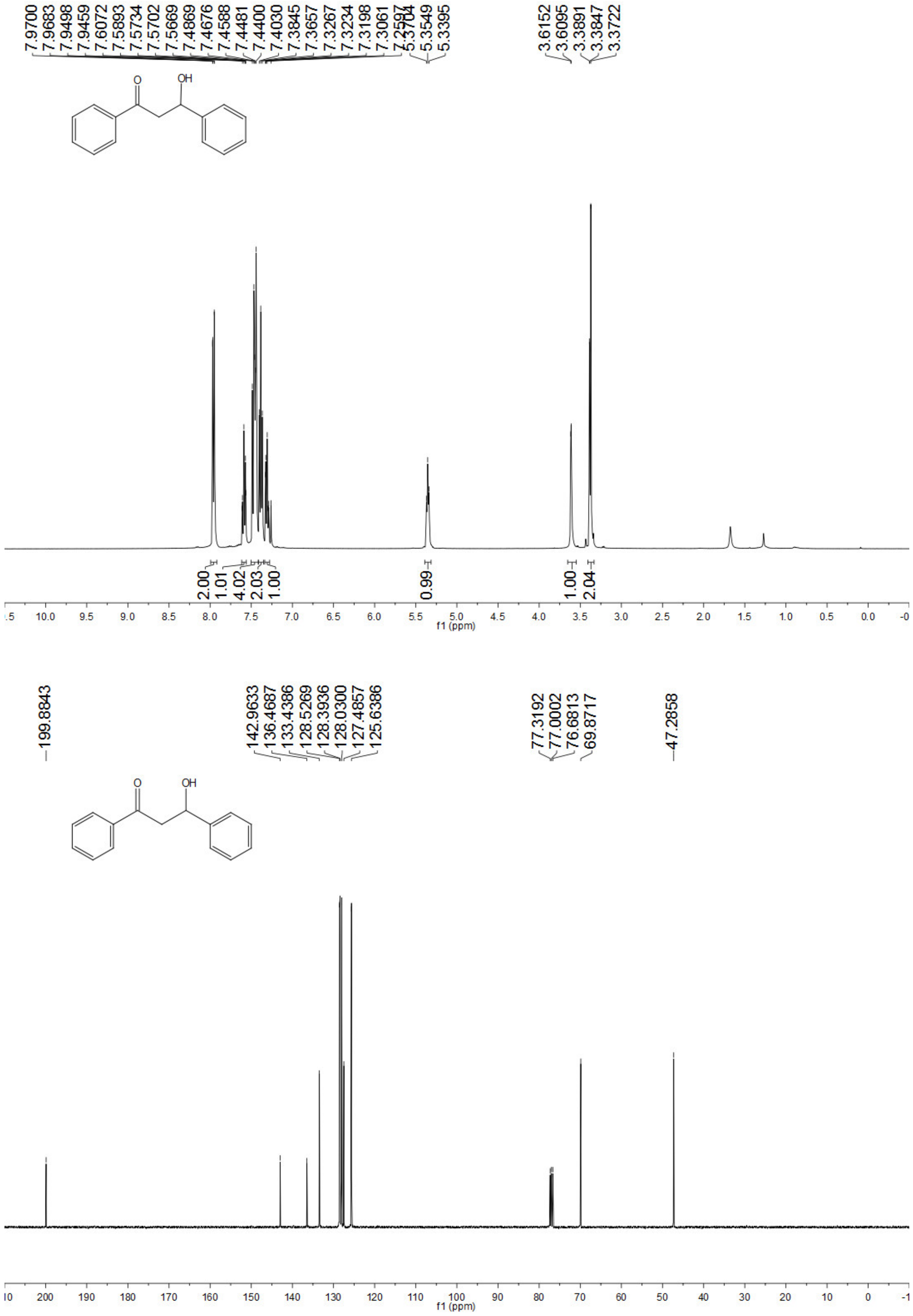

Figure S10. ^{1}H NMR and ^{13}C NMR spectra of **3h.**

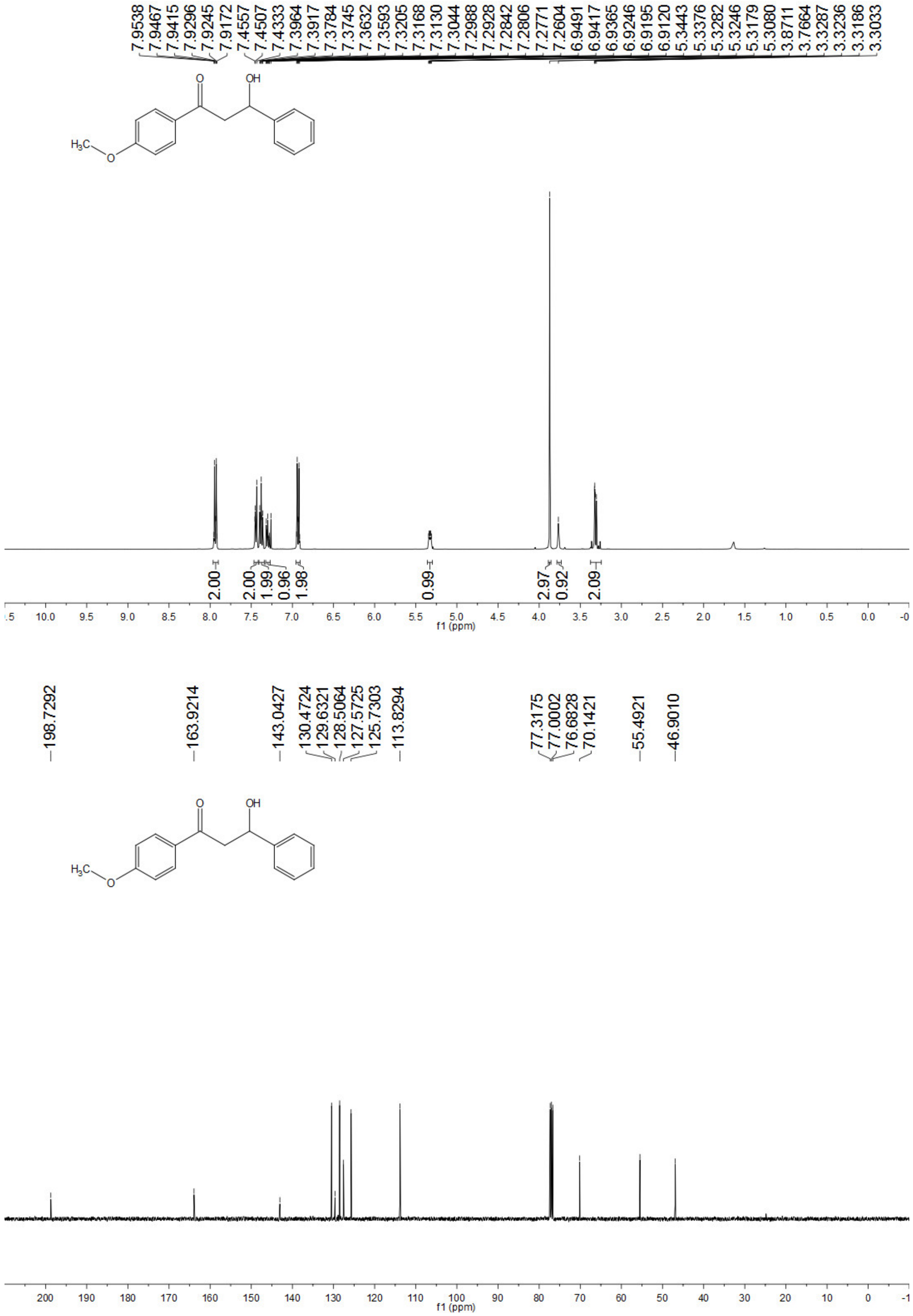

Figure S11. ^{1}H NMR and ^{13}C NMR spectra of **3i.**

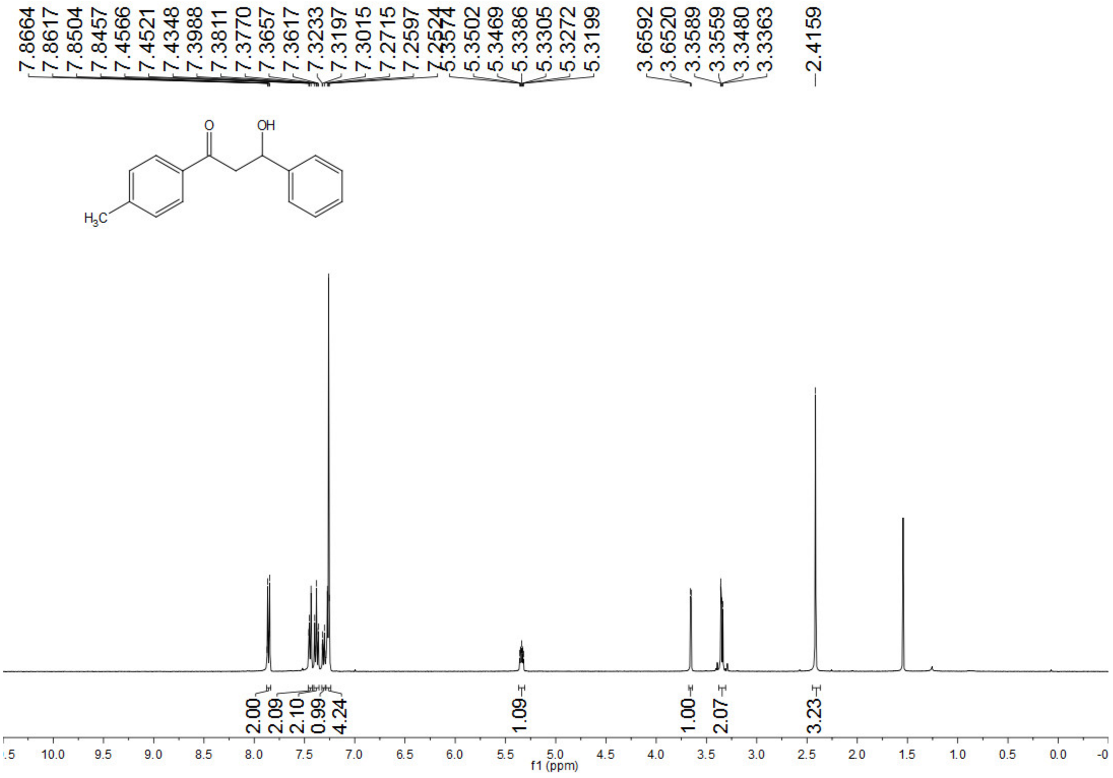

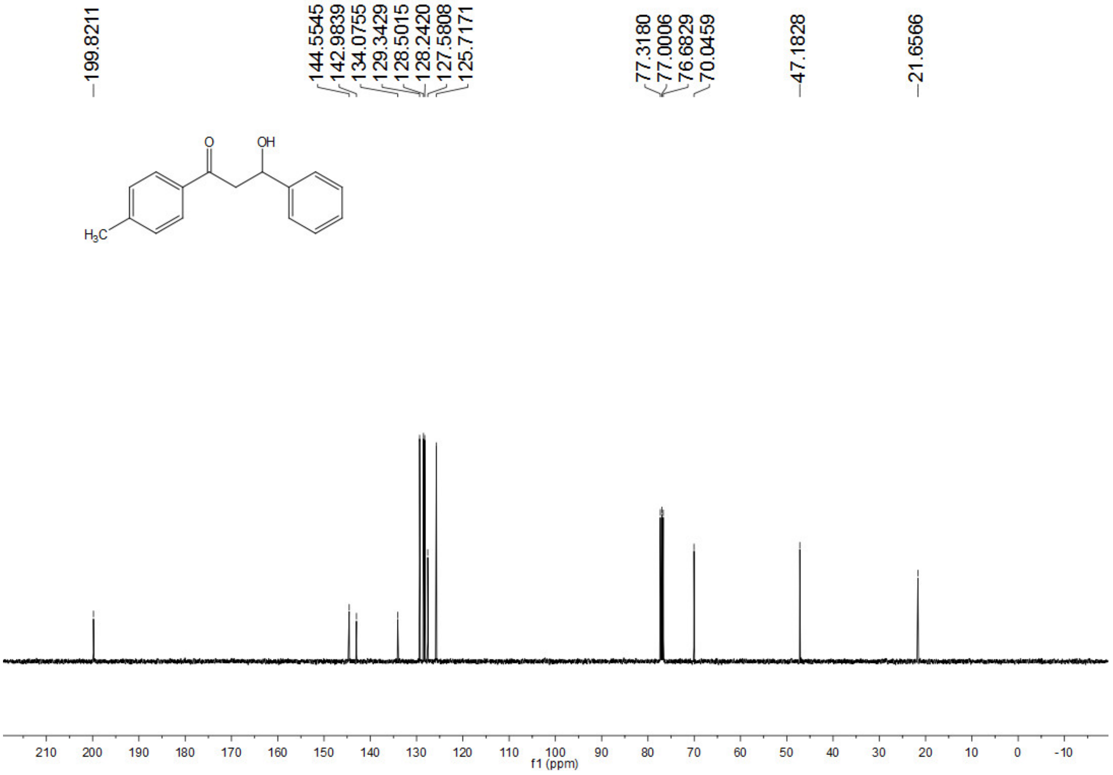

Figure S12. ^{1}H NMR and ^{13}C NMR spectra of **3j.**

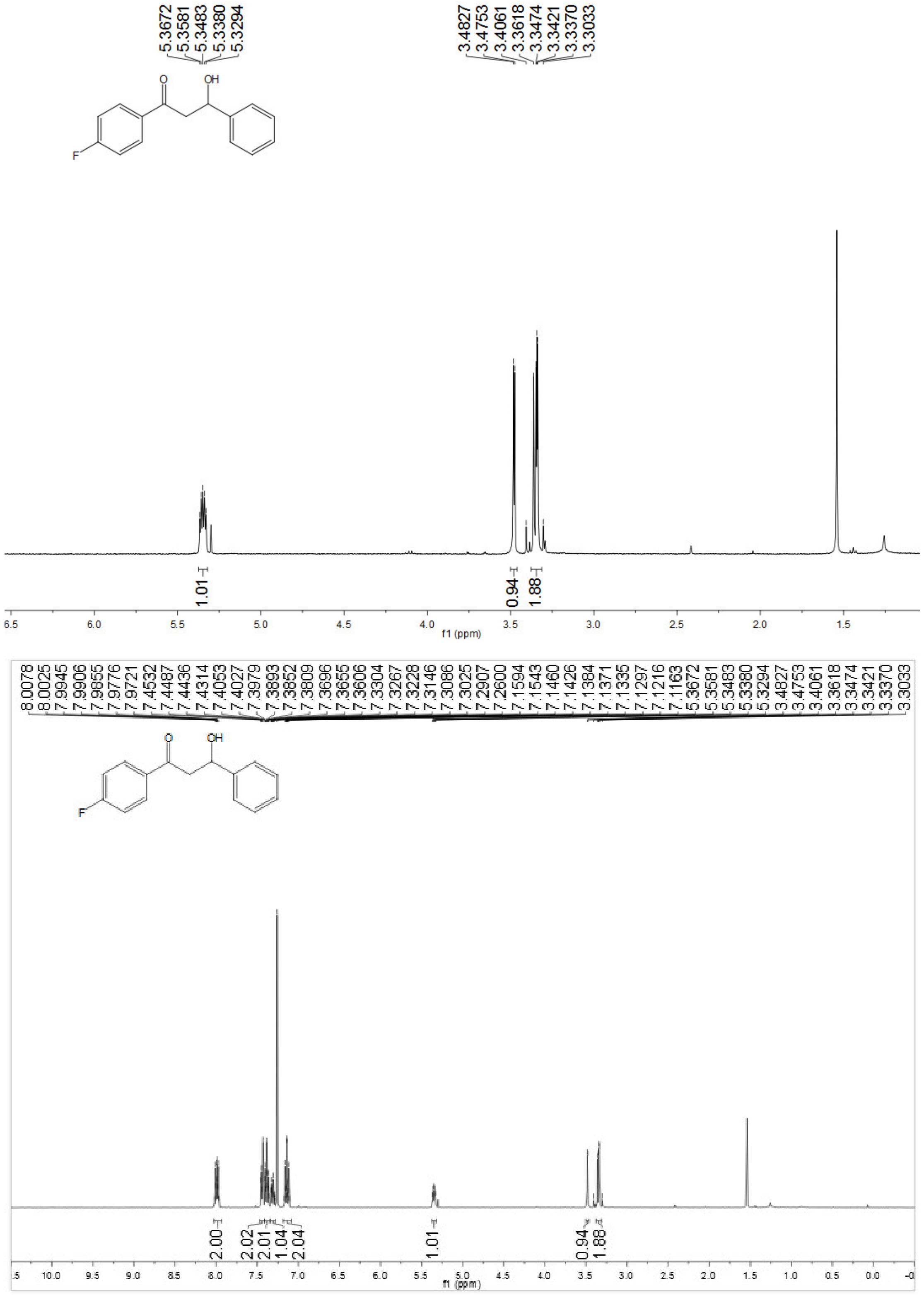

Figure S13. ^{1}H NMR and ^{13}C NMR spectra of **3k.**

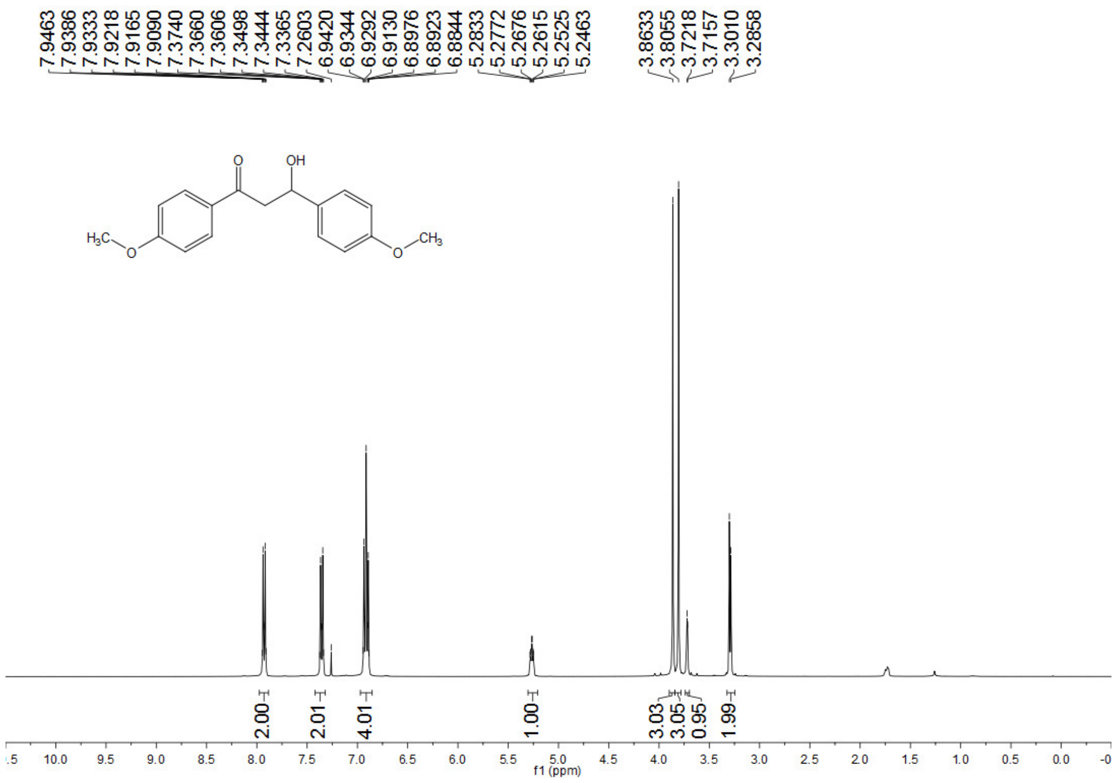

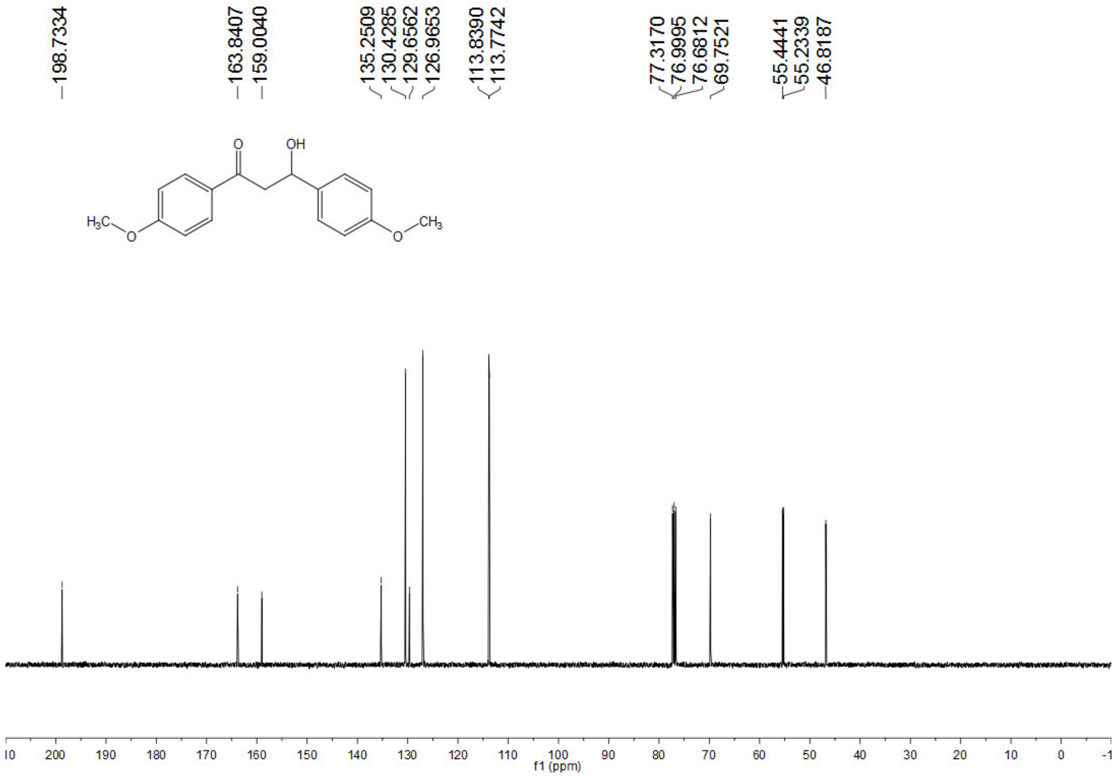

Figure S14. ^{1}H NMR and ^{13}C NMR spectra of **3l.**

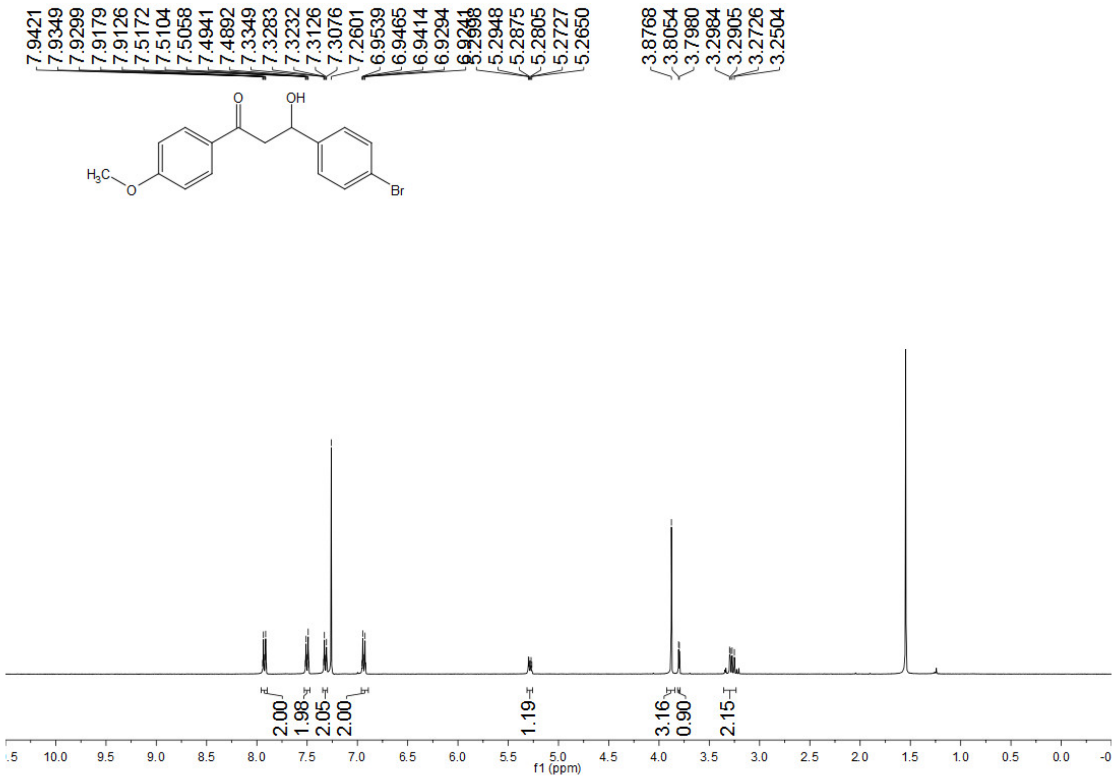

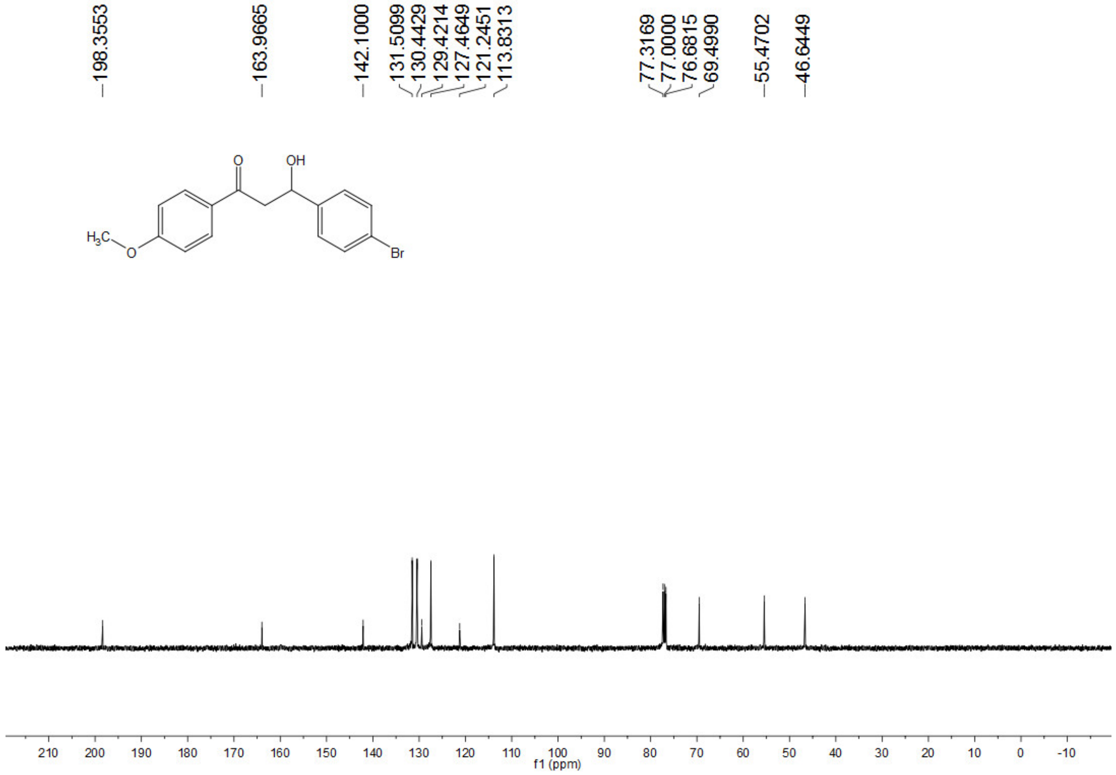

Figure S15. ^{1}H NMR and ^{13}C NMR spectra of **3m.**

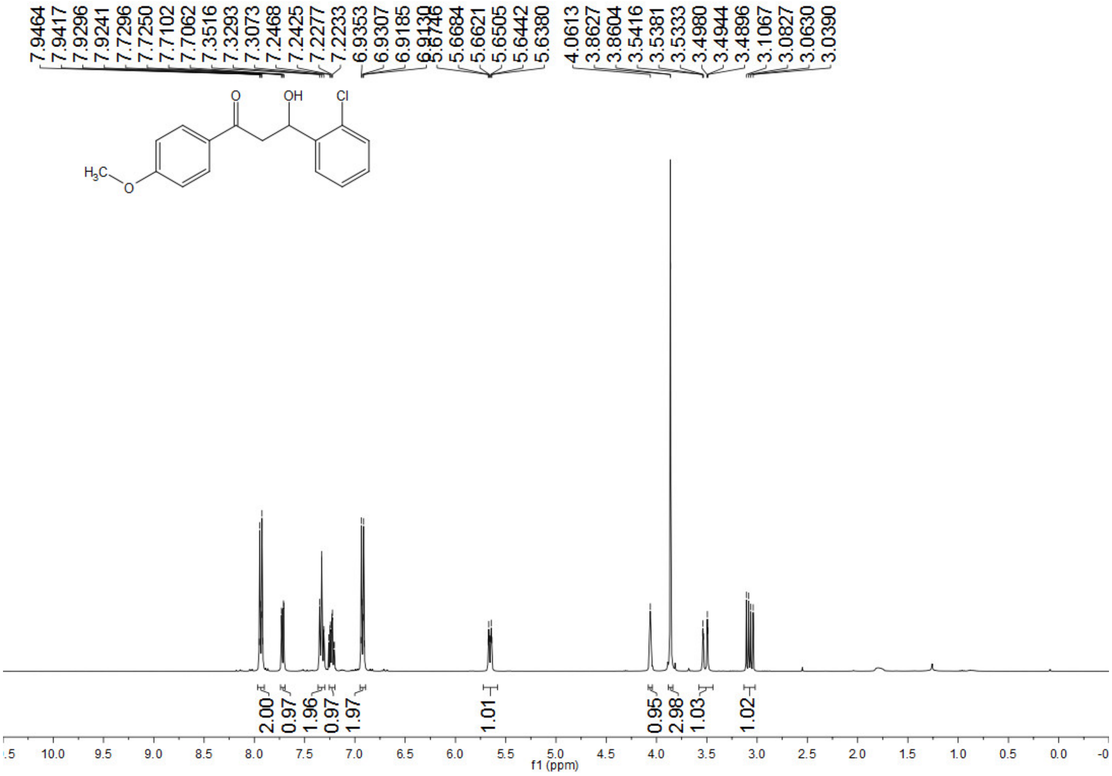

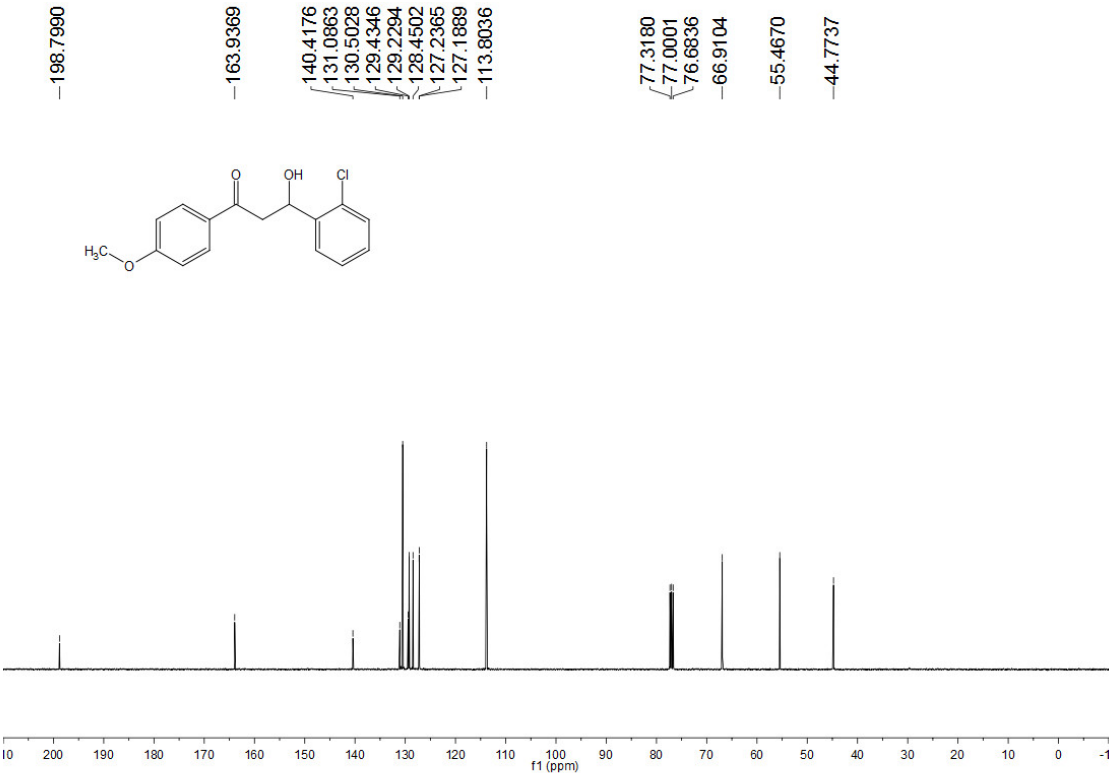

Figure S16. ^{1}H NMR and ^{13}C NMR spectra of **3n.**

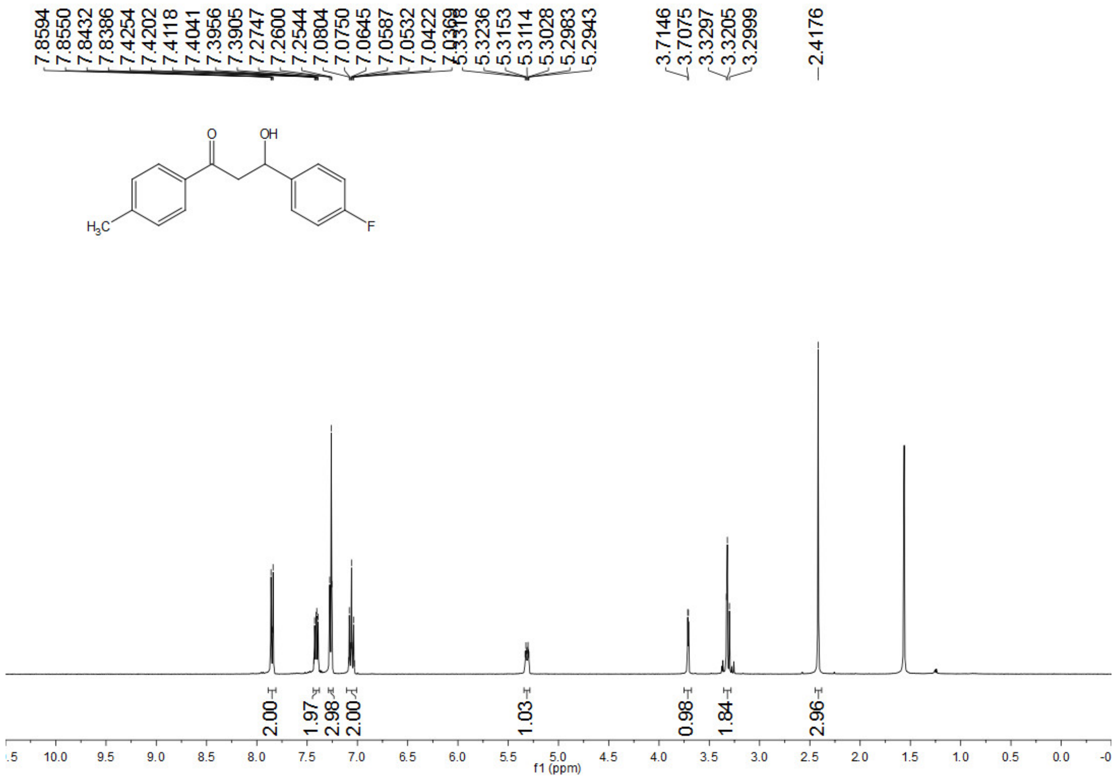

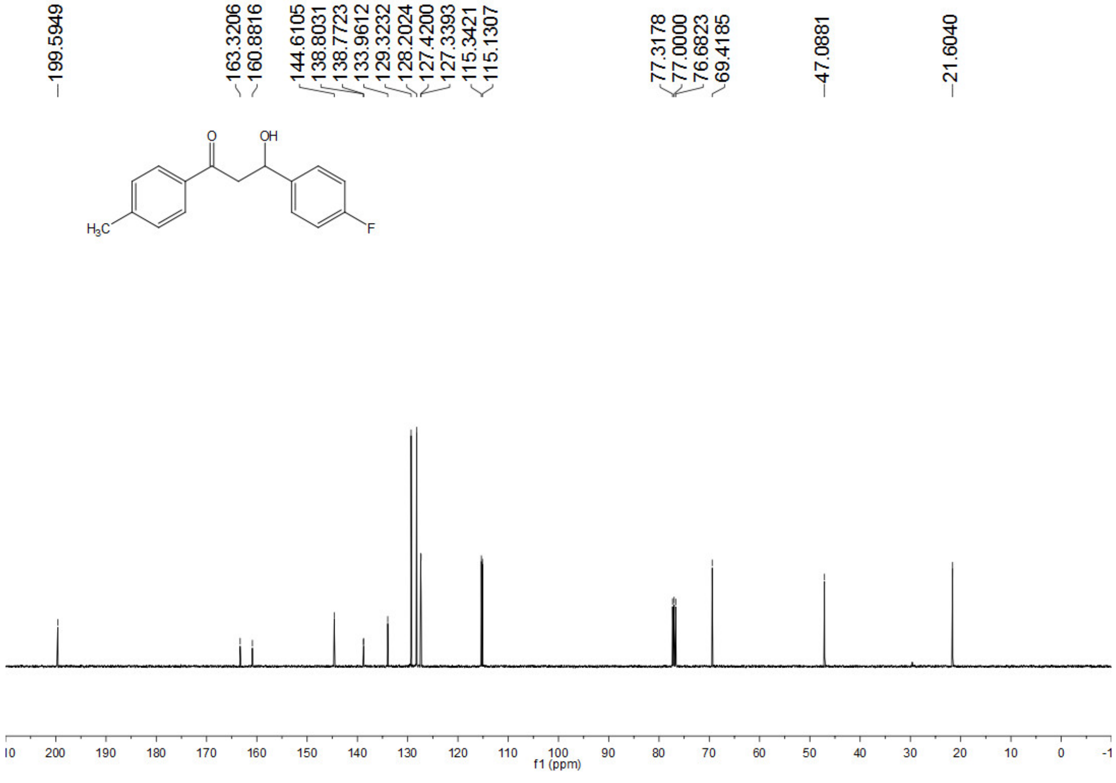

Figure S17. ^{1}H NMR and ^{13}C NMR spectra of **3o.**

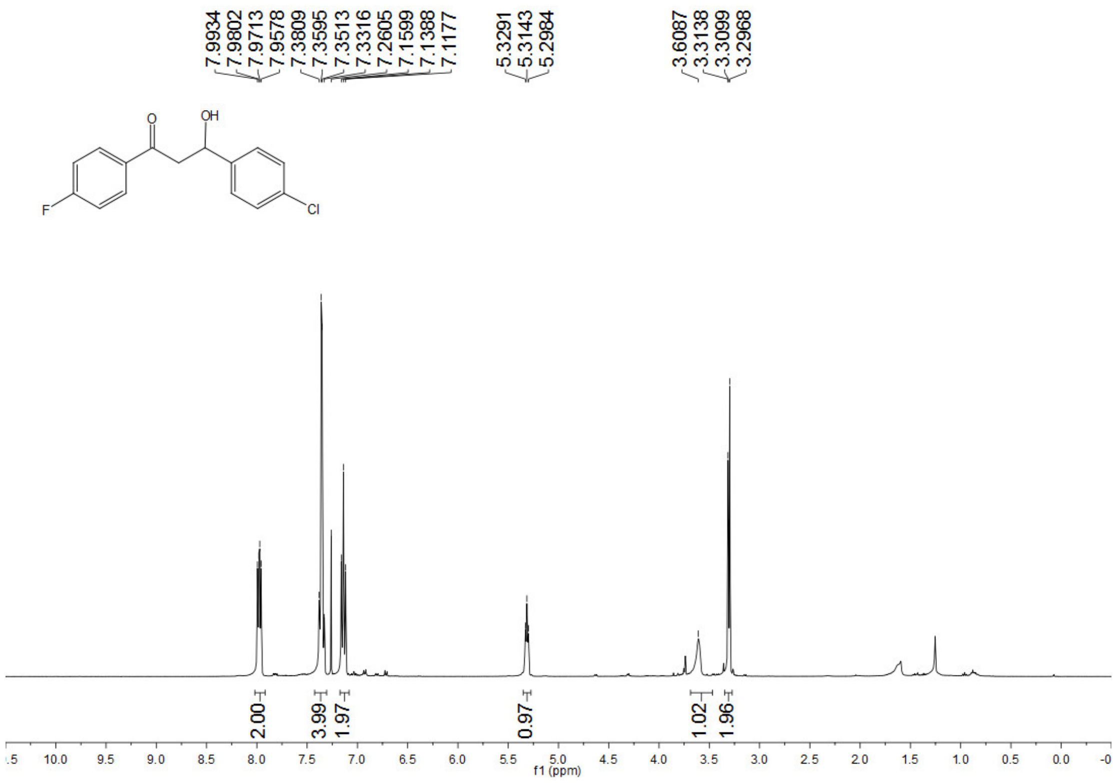

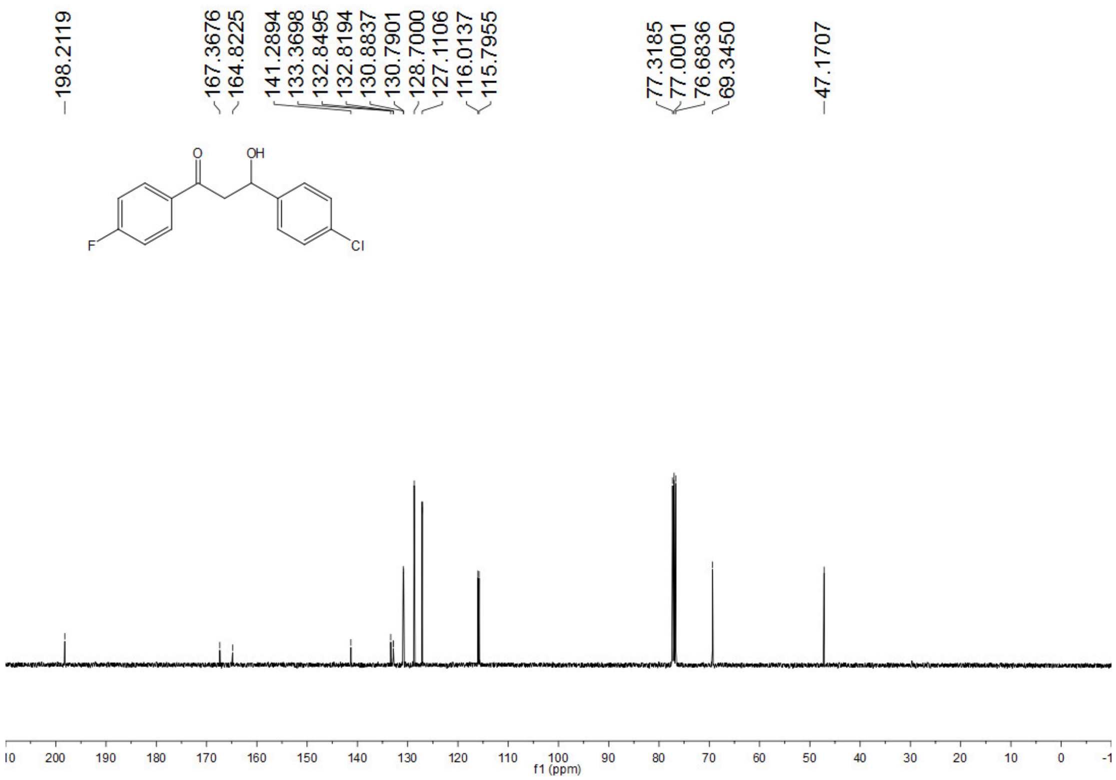

Figure S18. ^{1}H NMR and ^{13}C NMR spectra of **3p.**

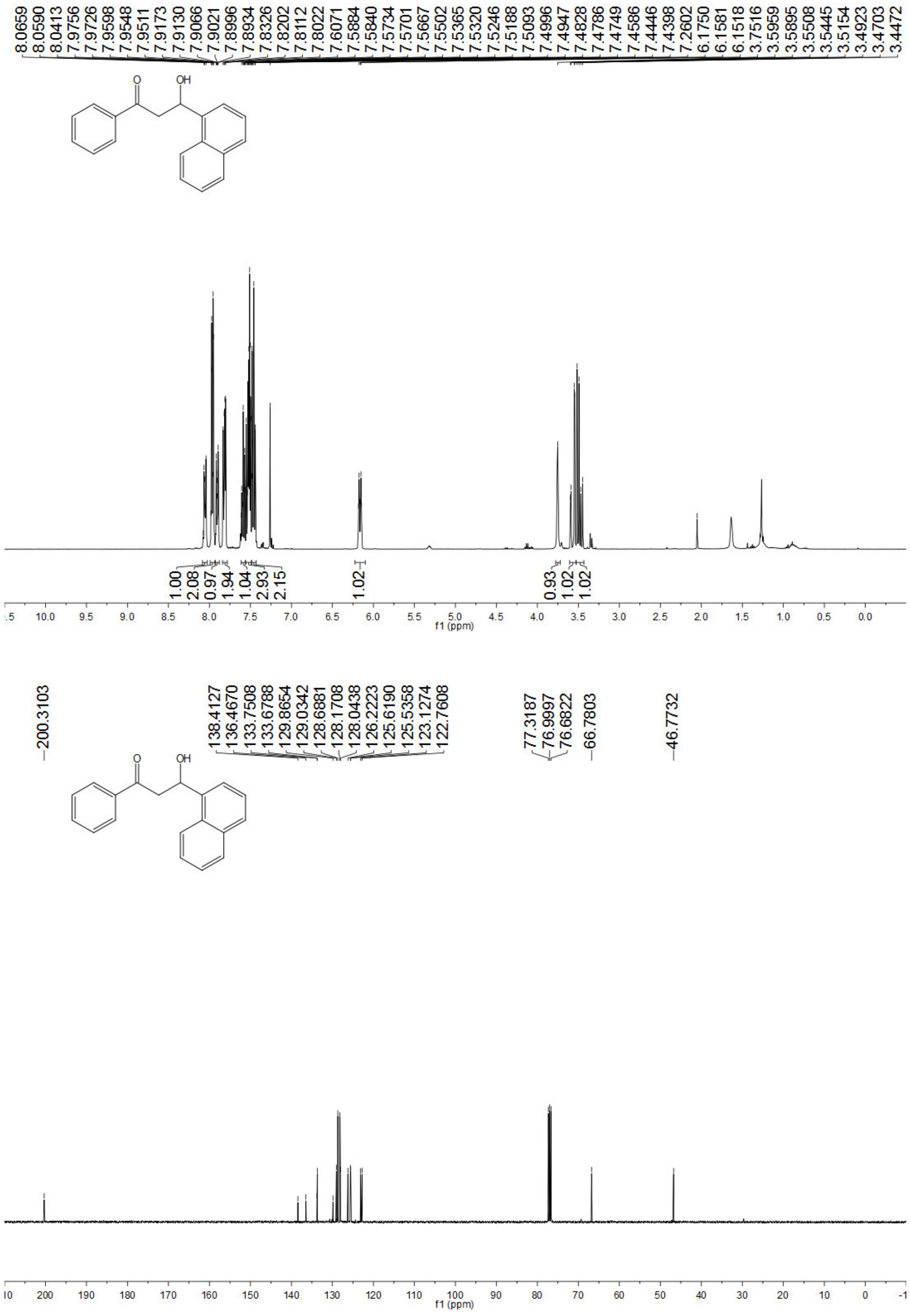

Figure S19. ^{1}H NMR and ^{13}C NMR spectra of **3q.**

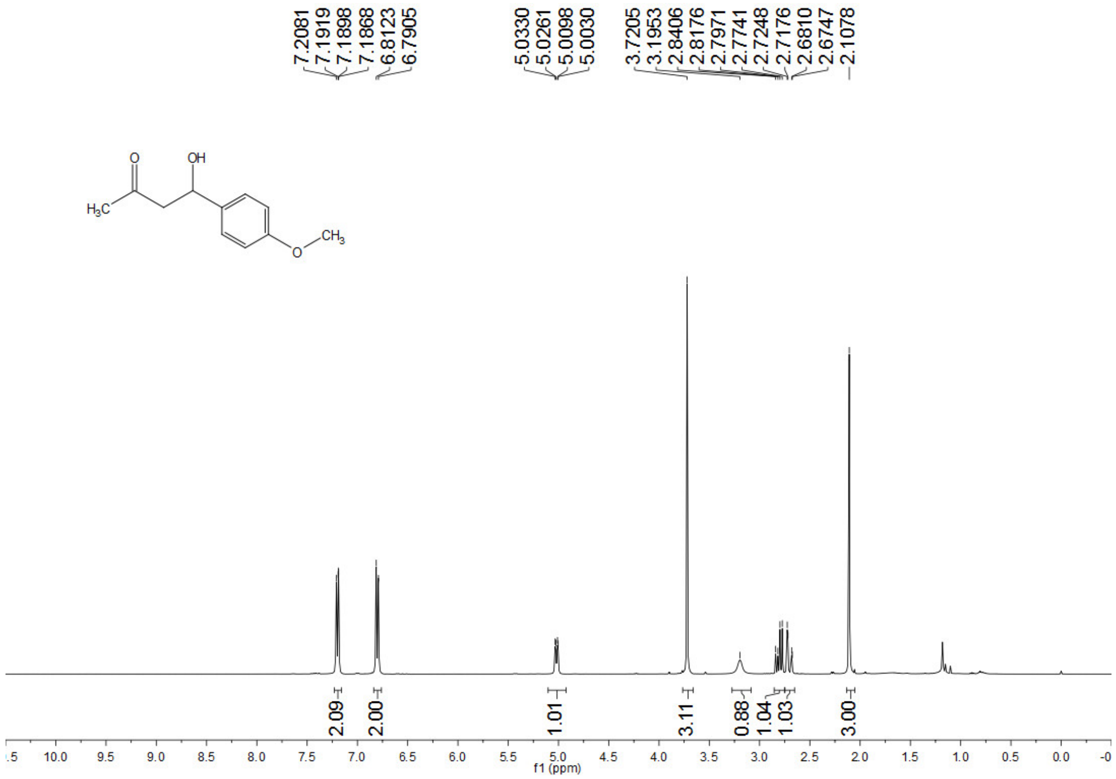

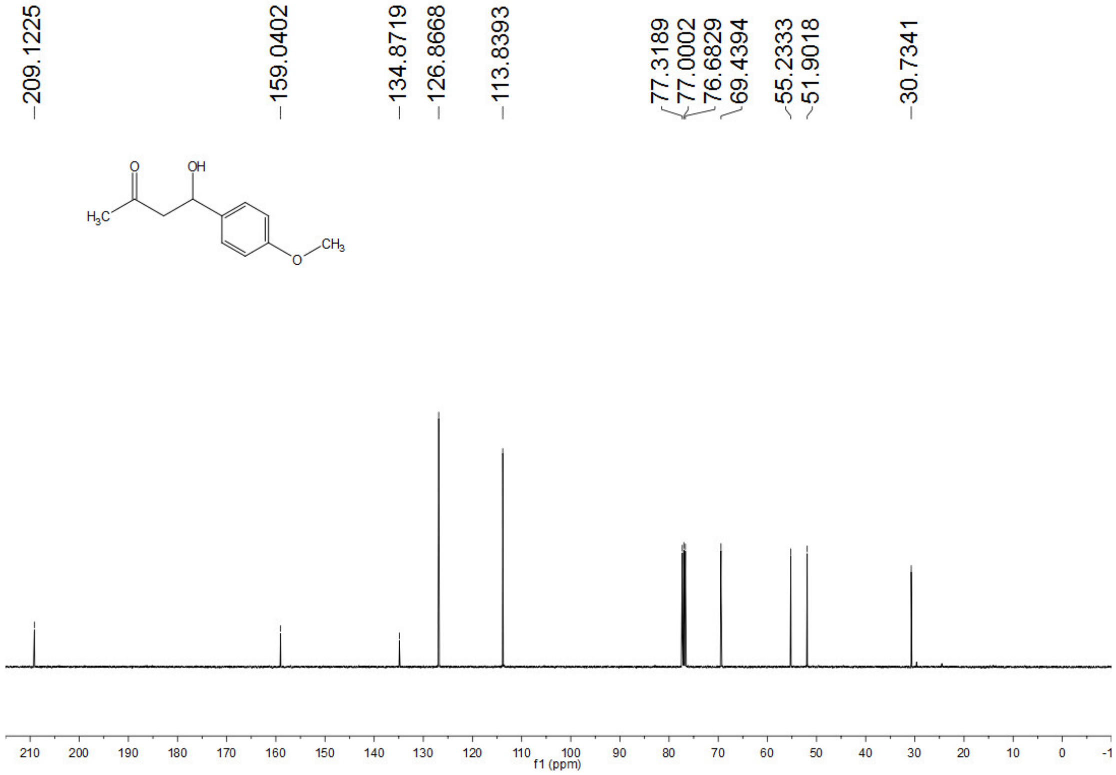

Figure S20. ^{1}H NMR and ^{13}C NMR spectra of **3r.**

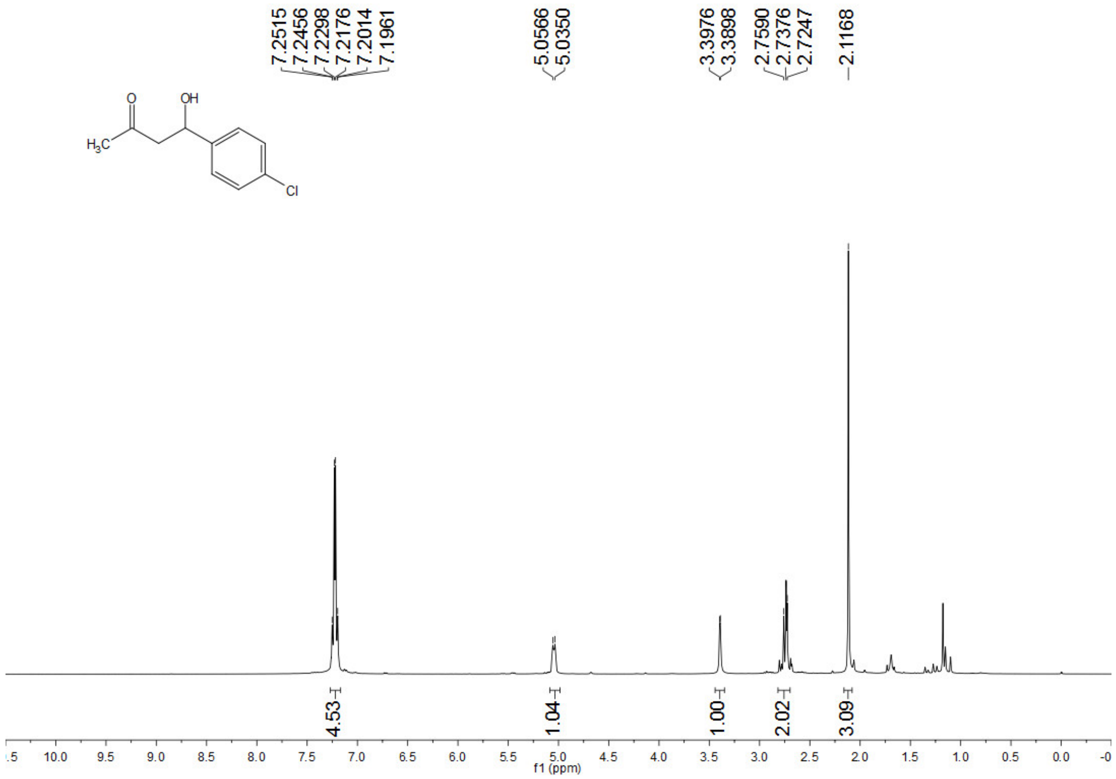

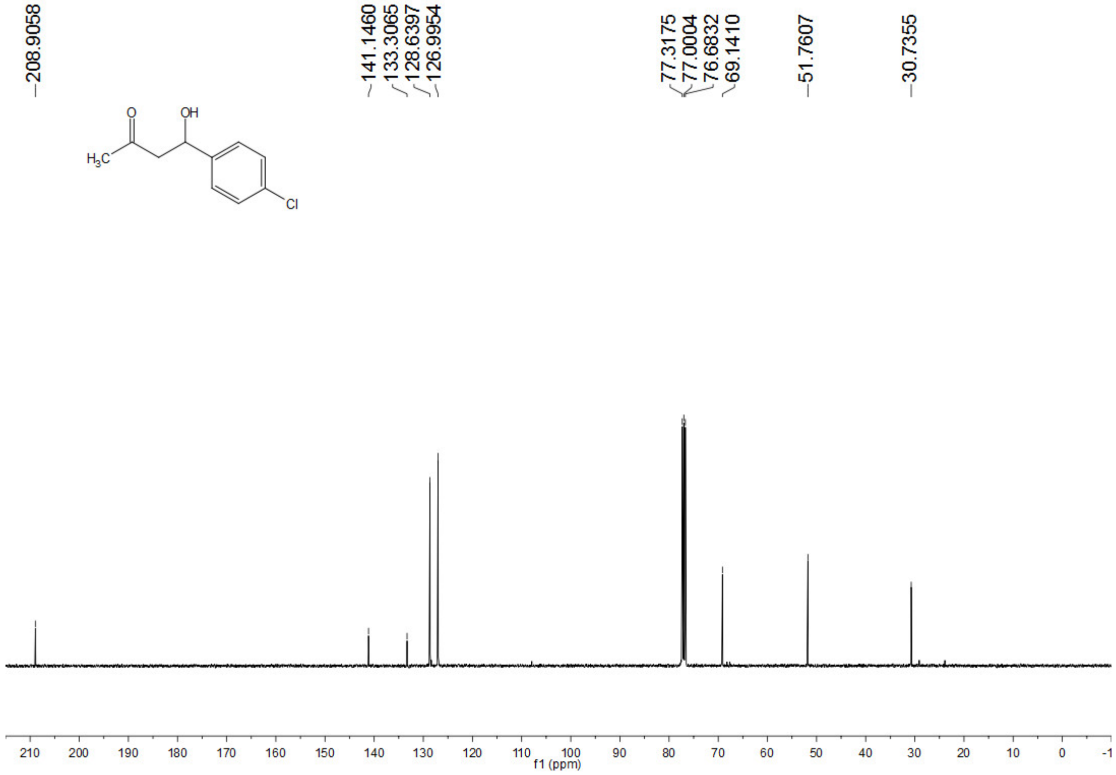

Figure S21. ¹H NMR and ¹³C NMR spectra of **3s.**

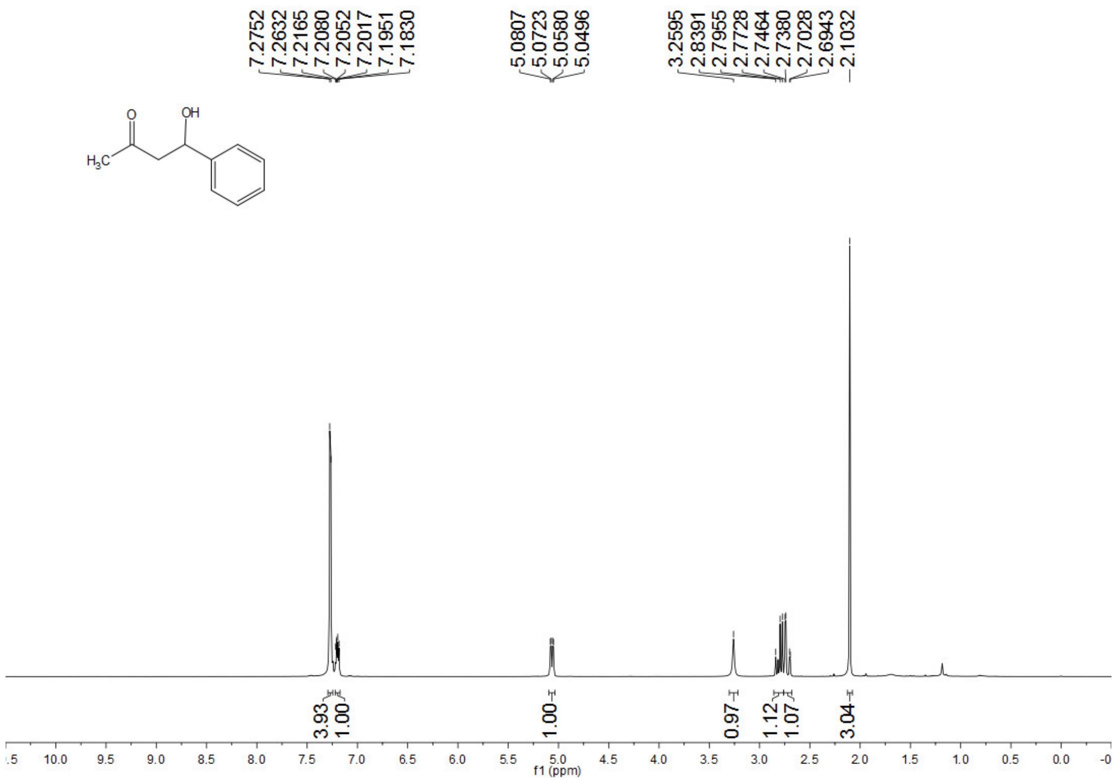

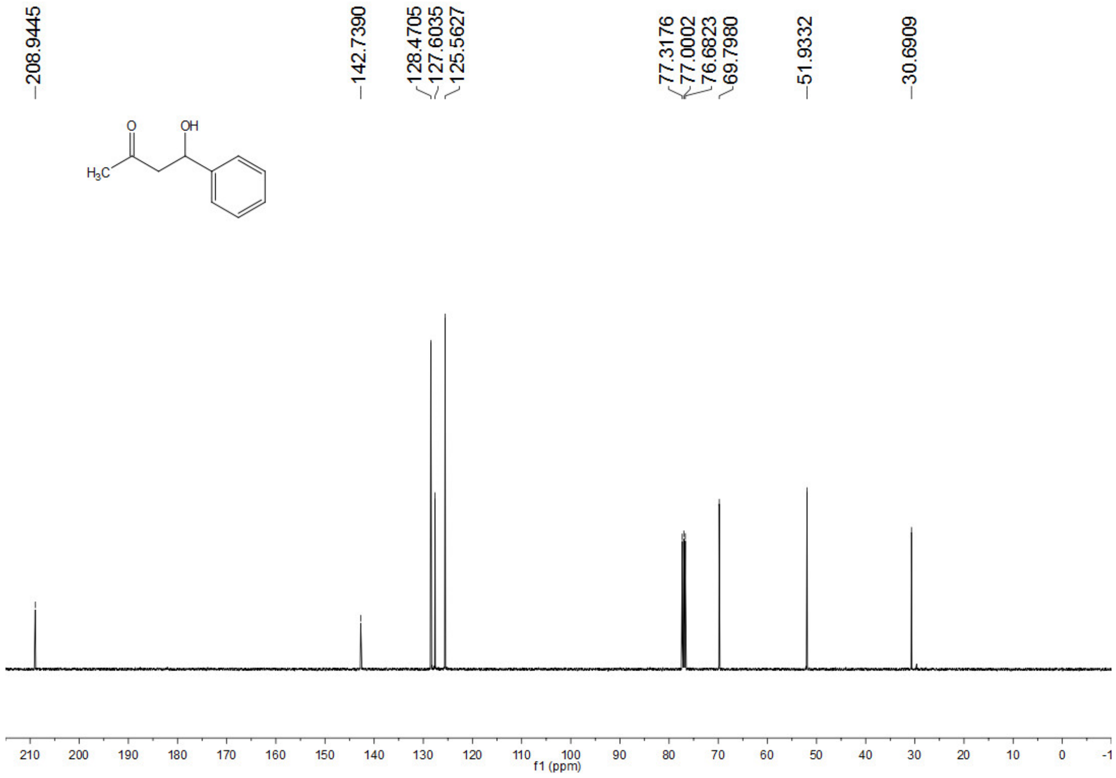

Figure S22. ¹H NMR and ¹³C NMR spectra of **3t.**

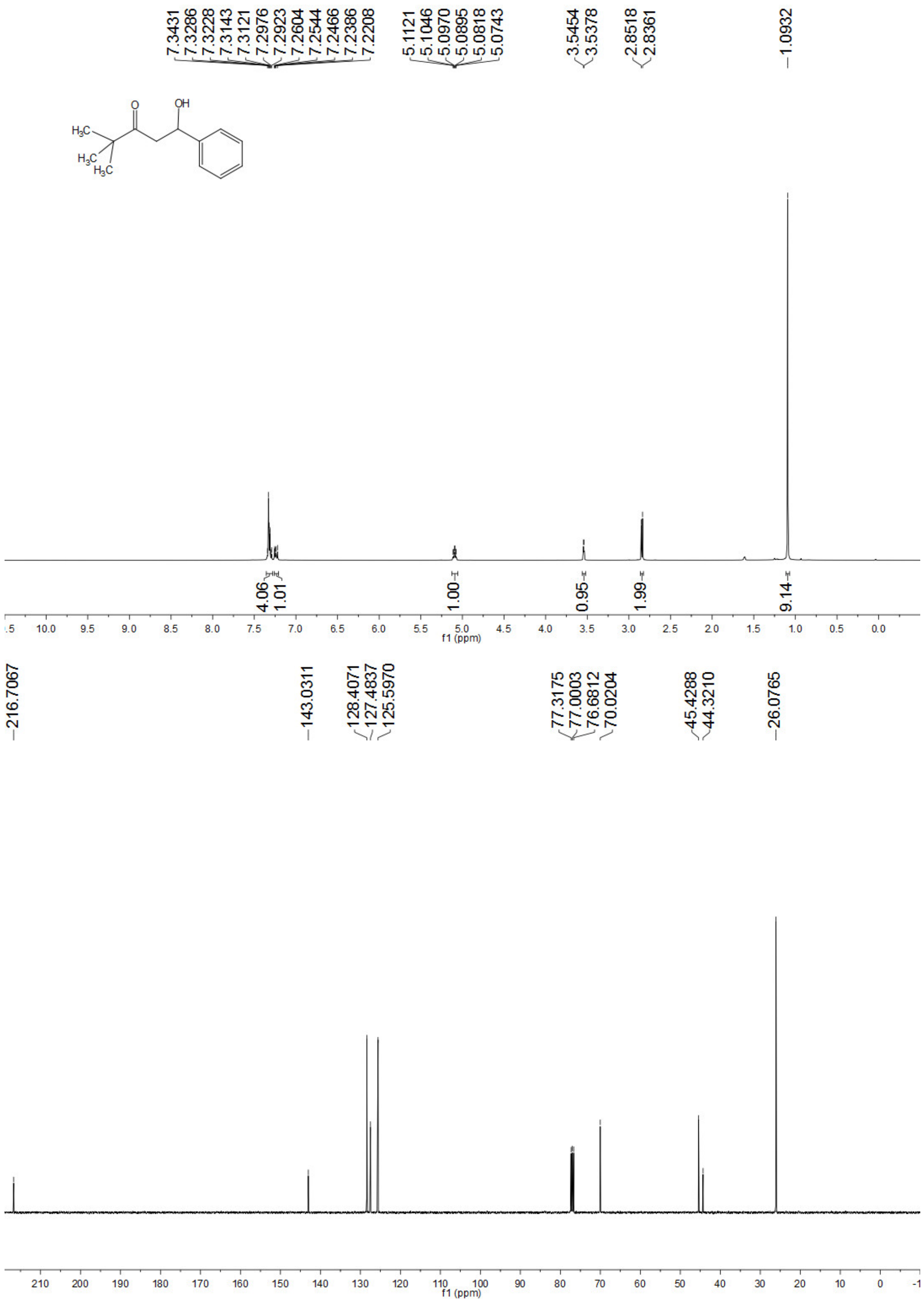

Figure S23. ^{1}H NMR and ^{13}C NMR spectra of **3u.**

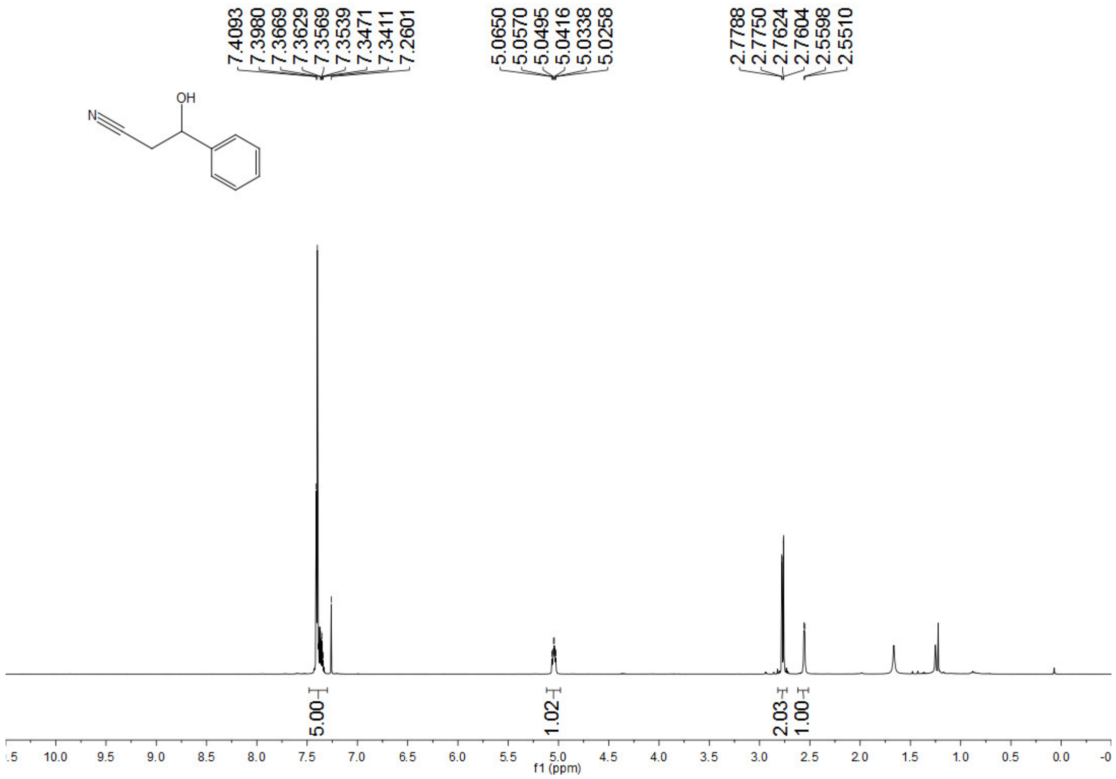

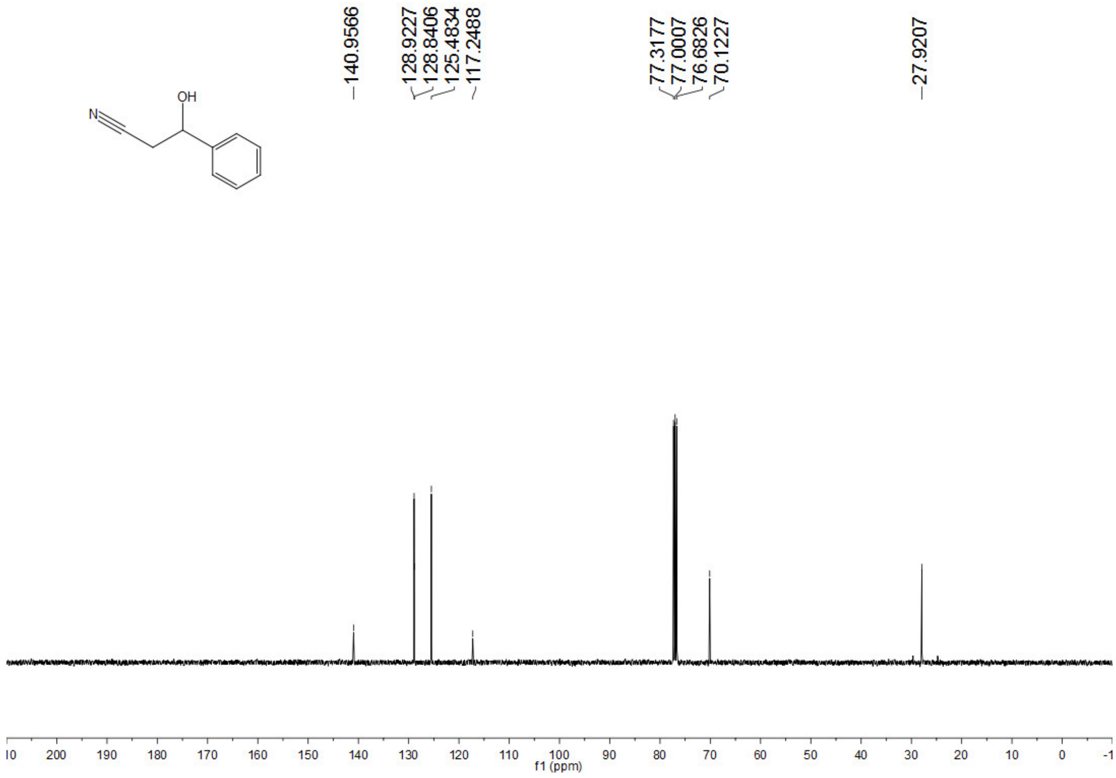

Figure S24. ^{1}H NMR and ^{13}C NMR spectra of **3v.**

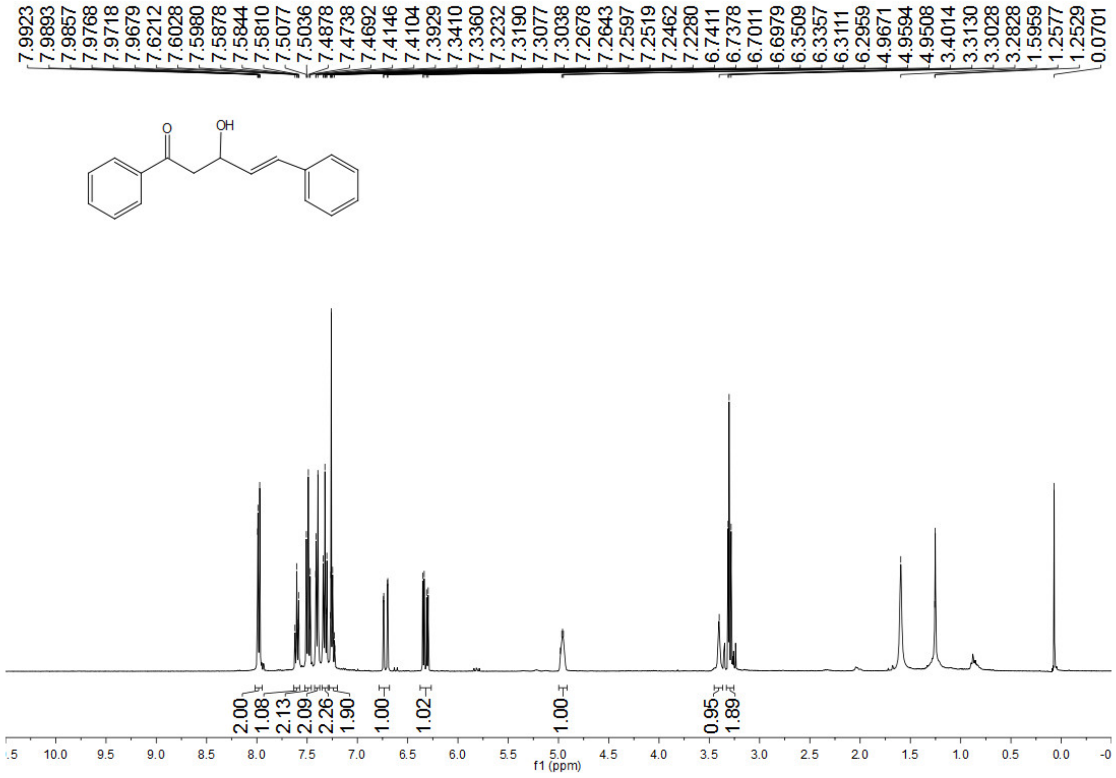

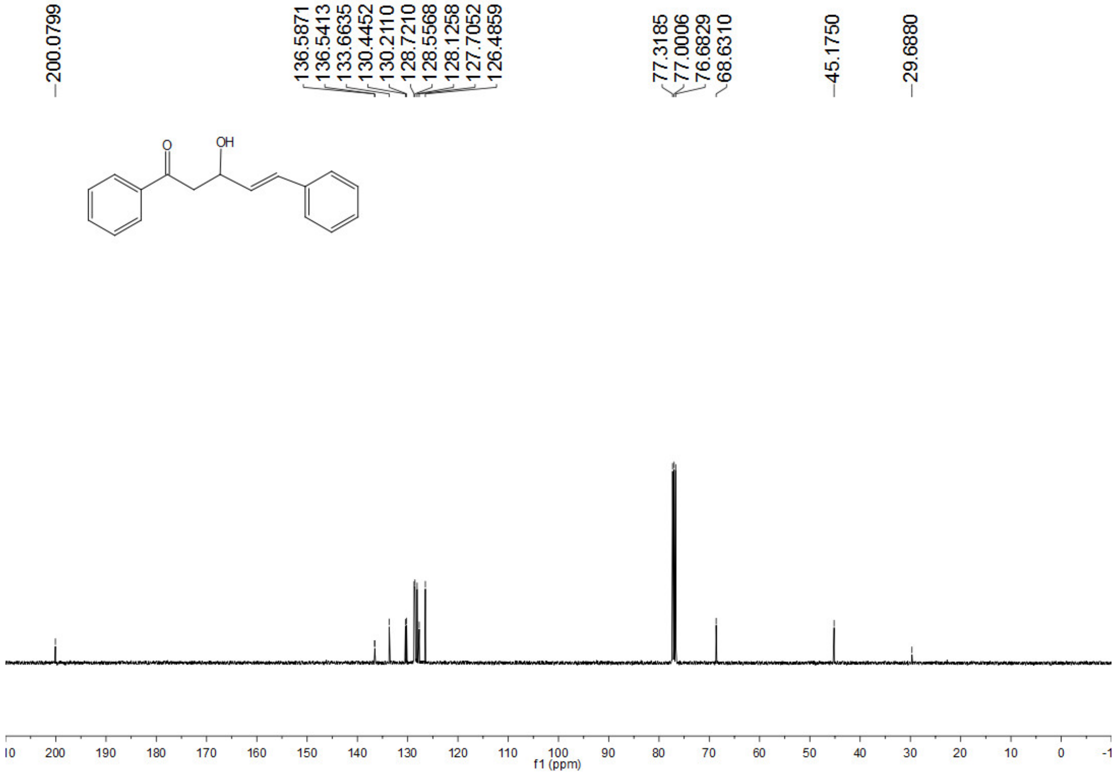

Figure S25. ^{1}H NMR and ^{13}C NMR spectra of **3w.**

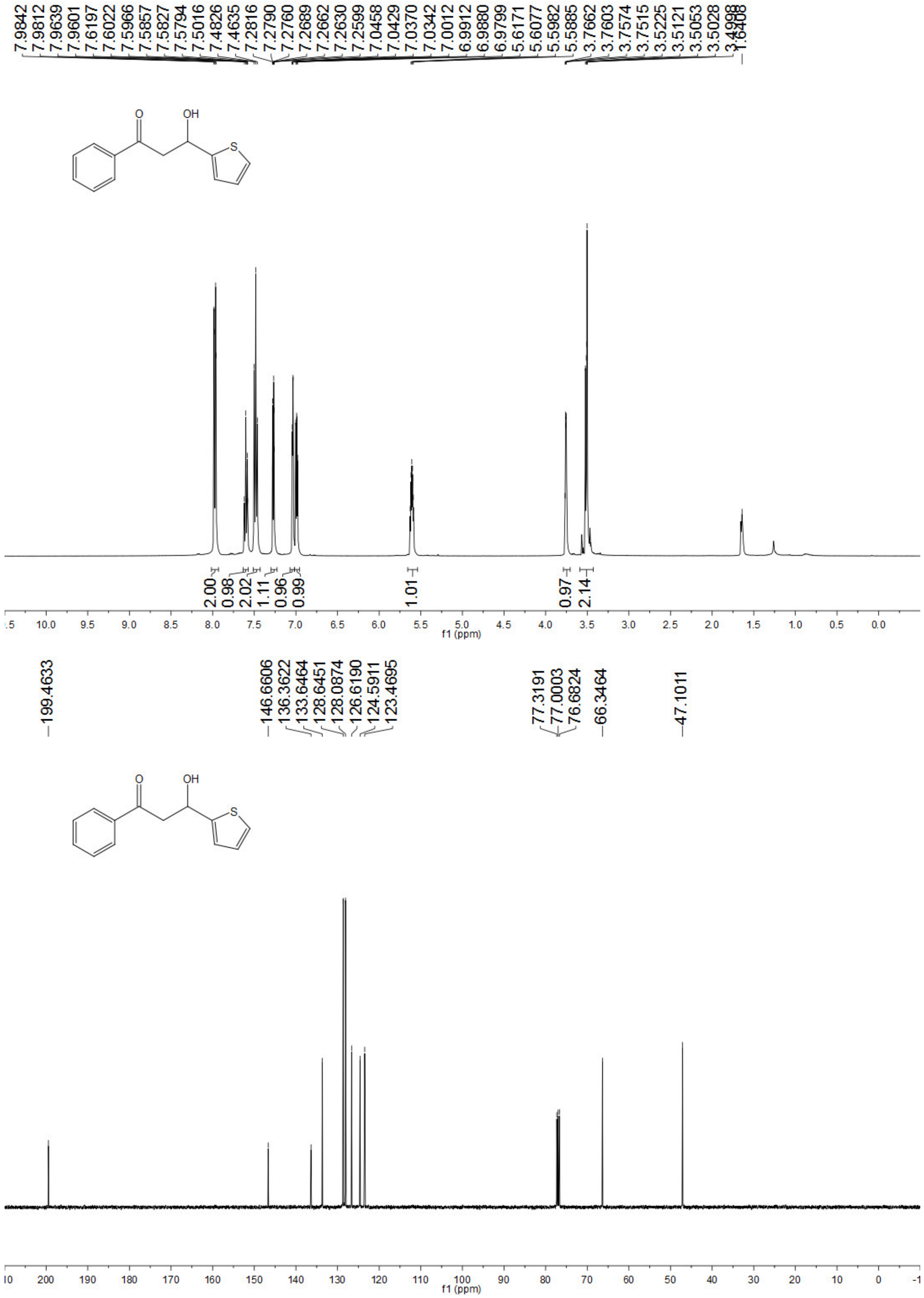

Figure S26. ^{1}H NMR and ^{13}C NMR spectra of **3x.**

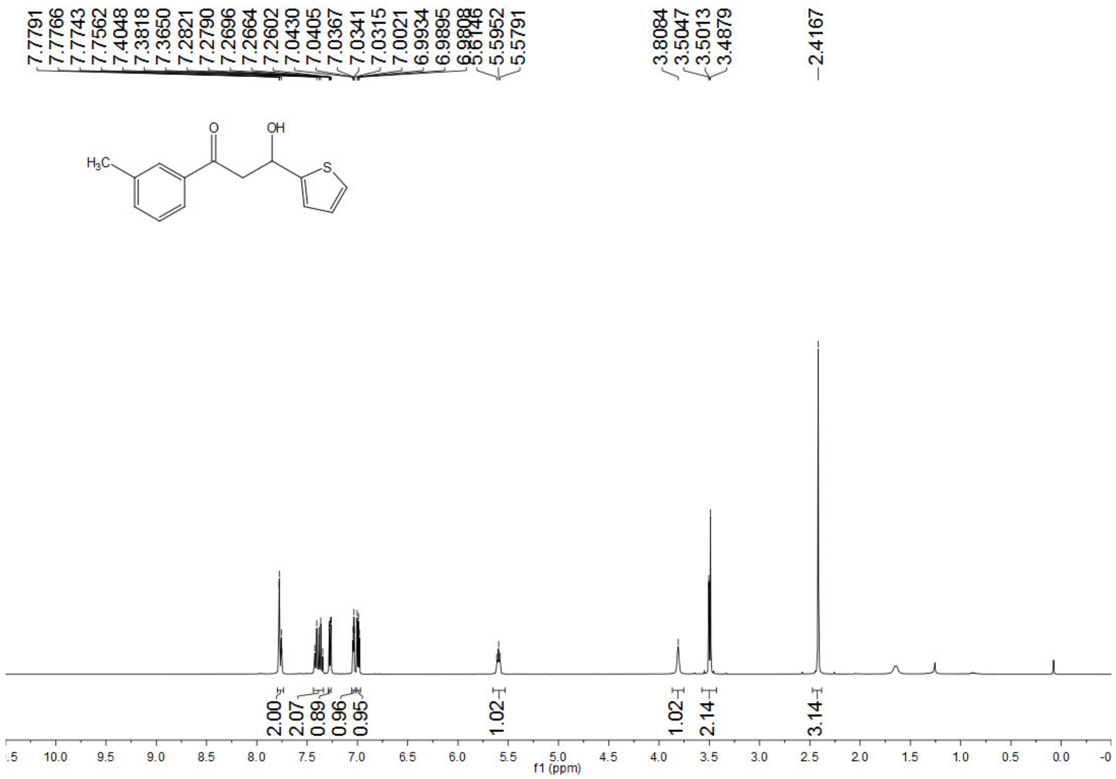

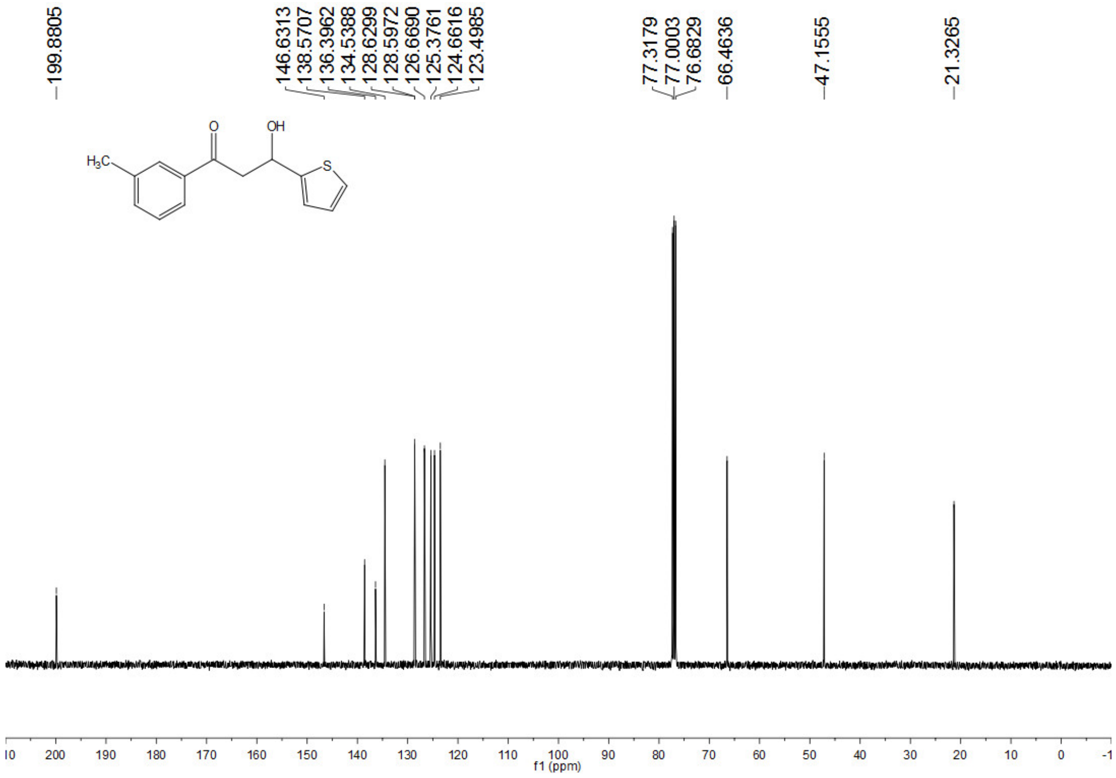

Figure S27. ^{1}H NMR and ^{13}C NMR spectra of **3y.**
